# Conserved roles of ETT and ARF4 in gynoecium development in Brassicaceae with distinct fruit shapes

**DOI:** 10.1242/dev.204263

**Published:** 2025-02-12

**Authors:** Heather Marie McLaughlin, Tian-Feng Lü, Bhavani Natarajan, Lars Østergaard, Yang Dong

**Affiliations:** ^1^ Department of Crop Genetics, The John Innes Centre, Norwich Research Park, Colney Lane, Norwich NR4 7UH, UK; ^2^ Sainsbury Laboratory at Cambridge University, 47 Bateman Street, Cambridge CB2 1LR, UK; ^3^ State Key Laboratory of Plant Diversity and Specialty Crops, Institute of Botany, Chinese Academy of Sciences, 20 Nanxincun, Xiangshan, Beijing 100093, China; ^4^ China National Botanical Garden, Beijing 100093, China; ^5^ Department of Biology, University of Oxford, South Parks Road, Oxford OX1 3RB, UK; ^6^ University of Chinese Academy of Sciences, Beijing 100049, China

**Keywords:** Gynoecium, Fruit, Auxin, Polarity, Morphogenesis, Signalling

## Abstract

Gynoecium patterning is dependent on the dynamic distribution of auxin, the signalling of which is transduced through several distinct pathways. ETTIN (ETT)-mediated signalling occurs independently of the canonical auxin pathway, and ETT shares partial redundancy with Auxin Response Factor 4 (ARF4) in the gynoecium. ETT and ARF4 were previously hypothesized to translate auxin gradients into patterns of tissue polarity alongside other ARFs. As ARF repressors, ETT/ARF were assumed to antagonistically regulate targets shared with ARF activators of the canonical pathway. Here, comparative transcriptomics identified the distinct and overlapping targets of ETT/ARF4 in the *Arabidopsis* gynoecium. However, ETT/ARF4 targets with known roles in gynoecium development did not conform to models of A-B ARF antagonism, leaving the relationship with the canonical pathway unclear. Mutants in *tir1 afb2 ett* were therefore generated in *Arabidopsis* and *Capsella* to assess the relationship between the two pathways, and their conservation in species with distinct fruit shapes. The data presented indicate conserved synergism between the two pathways in gynoecium development and suggest a role for ARF4 in the integration of these pathways in Brassicaceae with distinct fruit shapes.

## Teaser

**Summary:** An investigation of the relationship between ETT and ARF4, and canonical and ETT-mediated auxin signalling machineries in *Arabidopsis* and *Capsella* reveals conserved functions in *Brassicaceae* with distinct fruit shapes.

## INTRODUCTION

The gynoecium is the female reproductive organ produced in flowering plants that develops into the fruit after fertilization. Produced in the inner-most whorl of the flower, the gynoecium is composed of several specialized tissues along the apical-basal axis: the stigma, style and ovary (Sessions and Zambryski, 1995). In Brassicaceae, the ovary is made up of two valves, which are separated from one another by the valve margins and medial replum. Gynoecium patterning and morphogenesis are dependent on the dynamic distribution of the phytohormone auxin, which promotes both valve elongation and radialization of the style (Moubayidin and Østergaard, 2014; Dong and Østergaard, 2019).

Auxin signalling occurs via several distinct pathways in *Arabidopsis* (Dharmasiri et al., 2005; Kepinski and Leyser, 2005; Simonini et al., 2016; Fendrych et al., 2018; Cao et al., 2019; Kuhn et al., 2020; Friml et al., 2022). The canonical auxin signalling pathway induces the expression of auxin-sensitive genes in the nucleus (Paponov et al., 2008) and is composed of three main components: Auxin/Indole-3-Acetic Acid repressor proteins (AUX/IAAs), Transport Inhibitor Response1/Arabidopsis F-box (TIR1/AFB) receptors and Auxin Response Factors (ARFs). In the absence of auxin, ARFs are bound by AUX/IAAs, which recruit the TOPLESS (TPL) co-repressor, preventing the expression of auxin-responsive genes (Szemenyei et al., 2008). Auxin increases the affinity of the TIR1/AFBs for AUX/IAAs. AUX/IAAs are subsequently degraded by the proteasome, allowing ARFs to promote auxin responsive gene expression (Dharmasiri et al., 2005; Kepinski and Leyser, 2005).

ARFs are classified into three distinct clades: A-ARFs, B-ARFs and C-ARFs (Ulmasov et al., 1999; Tiwari et al., 2003; Finet et al., 2013). A-ARFs are transcriptional activators that readily interact with AUX/IAAs of the canonical pathway, while B and C ARFs are characterized as repressive, and interact with AUX/IAAs less readily (Ulmasov et al., 1999; Tiwari et al., 2003; Finet et al., 2013; Vernoux et al., 2011; reviewed by Guilfoyle and Hagen, 2012). It has previously been shown that A and B ARFs antagonistically regulate shared targets in *Marchantia* (Kato et al., 2020) and *Physcomitrium* (Lavy et al., 2016).

The ETTIN (ETT/ARF3)-mediated pathway was elucidated more recently in the context of its role in gynoecium development (Simonini et al., 2016; Kuhn et al., 2020). ETT is a B-class repressive ARF that lacks the C-terminal domain required for AUX/IAA interaction (Simonini et al., 2016) and functions independently of the canonical pathway (Kuhn et al., 2020). ETT recruits a TPL-HISTONE DEACETYLASE 19 (HDA19) complex to prevent the transcription of target loci in the absence of auxin. ETT interacts with auxin through its C-terminal ETTIN-SPECIFIC (ES) domain and undergoes a conformational change upon auxin binding that breaks the ETT-TPL-HDA19 complex. Histones then become acetylated and genes are derepressed (Kuhn et al., 2020). This mode of gene regulation by ETT is consistent with A-B ARF antagonism.

*ett* mutants exhibit gynoecium defects that phenocopy aberrations produced upon treatment with the auxin transport inhibitor N-1-Naphthylphthalamic Acid (NPA), highlighting the importance of ETT-mediated signalling for auxin-related processes in carpel development (Nemhauser et al., 2000).

ETT functions partly redundantly with ARF4, another B-class ARF that can interact with AUX/IAAs via its C-terminal PB1 domain (Pekker et al., 2005; Kelley et al., 2012; Finet et al., 2010; Vernoux et al., 2011). While the transcriptional targets of ETT have been well characterized (Simonini et al., 2017; Andres-Robin et al., 2020), those of ARF4 have not.

Here, comparative transcriptomics analyses were conducted to identify distinct and overlapping ETT and ARF4 targets in *Arabidopsis*. The data presented suggest that the role of ETT and ARF4 are twofold: first, ETT and ARF4 redundantly regulate genes involved in cell fate specification independently of auxin, and the mis-regulation of these genes correlates with the phenotypes observed in the *Arabidopsis ett arf4* mutants. Second, ETT and ARF4 maintain the auxin insensitivity of a range of target loci, many of which became auxin sensitive upon their mutation, suggesting that the A-B ARF antagonism previously observed in *Marchantia* (Kato et al., 2020) and *Physcomitrium* (Lavy et al., 2016) may regulate a range of biological processes in *Arabidopsis.*

Interestingly (as ETT and ARF4 are both B-class repressive ARFs), none of the genes that exhibited expression patterns consistent with A-B ARF antagonism in this study have a known role in gynoecium development. However, previous studies have proposed that ETT and ARF4 may function with other ARFs to specify tissue identity in lateral organs in response to auxin gradients (Pekker et al., 2005).

In line with this hypothesis, the expression of several canonical auxin receptors – *TIR1*, *AFB1*, *AFB2* and *AFB3 –* has previously been observed in the developing gynoecium (Dharmasiri et al., 2005; Cecchetti et al., 2008). Higher order *tir1afb1245* mutants have been shown to exhibit curled siliques and reduced fertility, which are indicative of gynoecium defects (Prigge et al., 2020), and previous work has shown that other AUX/IAA-interacting ARFs (Vernoux et al., 2011; Piya et al., 2014) are also involved in the regulation of gynoecium development (Nagpal et al., 2005; Wang et al., 2005). We therefore hypothesize that the canonical auxin signalling pathway may function synergistically with ETT/ARF4 to promote gynoecium development.

To test this hypothesis, *ett-3* mutants were crossed with *tir1-1 afb2-3* canonical pathway receptor mutants in *Arabidopsis* to generate a triple mutant*.* Gynoecia of *tir1-1 afb2-3 ett-3* mutants showed exacerbation of the *ett-3* phenotype, suggesting that the canonical and ETT-mediated pathways function synergistically in gynoecium development. Furthermore, the *tir1-1 afb2-3 ett-3* triple mutant partially phenocopied the *ett-3 arf4-2* double mutant, suggesting a role for ARF4 in the integration of signalling between the canonical and ETT-mediated pathways.

The Brassicaceae family display an impressive array of fruit shape diversity including cylindrical, spherical, disc, and heart-shaped fruits (Langowski et al., 2016). Gynoecia from species producing highly divergent fruit shapes still maintain the same general organization of tissues, with two or more carpels and an apical radial style topped with stigmatic papillae (Langowski et al., 2016). Members of the Brassicaceae family therefore provide excellent models to study conserved mechanisms of carpel development. It was recently shown that gynoecium development in *Capsella rubella* (from here on referred to as *Capsella*), which diverged from *Arabidopsis* around 8 million years ago and produces heart shaped fruits, is also dependent on auxin dynamics throughout development (Dong et al., 2019).

To assess whether synergism between ETT-mediated and the canonical signalling machineries are conserved in species with distinct fruit shapes, *tir1 afb2 ett* mutants were also generated in *Capsella*. As in *Arabidopsis*, the *Capsella tir1 afb2 ett* triple mutant exacerbated the *ett* phenotype and phenocopied the *ett arf4* double mutant, suggesting that synergism between canonical and ETT-mediated signalling machineries is conserved, as is the role of ARF4 in the integration of auxin signalling between the two pathways.

## RESULTS

### Comparative transcriptomics in *Arabidopsis* dissect distinct and overlapping targets of ETT and ARF4 in gynoecium development

ETT is known to function partially redundantly with ARF4 in the regulation of gynoecium development (Pekker et al., 2005; Finet et al., 2010; Kelley et al., 2012). While ETT-mediated signalling and the regulation of ETT-target genes are relatively well studied in the gynoecium (Simonini et al., 2016, 2017; Kuhn et al., 2020), a global understanding of how ARF4 regulates its targets, and which genes it modulates in conjunction with ETT, is lacking.

To better understand how ARF4 regulates its targets both alone and together with ETT, a comparative transcriptomic analysis (RNA sequencing, from here on referred to as RNA-seq) was conducted using whole inflorescence tissues of *Arabidopsis* wild type (Col-0), *ett-3*, *arf4-2* and *ett-3 arf4-2* lines treated with 100 µM Indole-3-Acetic Acid (IAA) and 10 µM N-1-Naphthylphthalamic Acid (NPA) or mock treatments for 1 h.

A principal component analysis revealed that the lines differed more by genotype than by auxin treatment (Fig. S1), and many genes that are reportedly auxin responsive (Paponov et al., 2008) were modulated in response to auxin in wild-type plants, indicating that the auxin treatment was effective (Table S1). *PINOID*, a previously well-characterized ETT target (Simonini et al., 2016, 2017; Kuhn et al., 2019) that is upregulated in wild type in response to auxin (Kuhn et al., 2020), was not identified as an auxin-sensitive gene within this study. Previous transcriptomics analyses aiming to identify ETT targets used different schedules of auxin treatment (Kuhn et al., 2020; Simonini et al., 2017), which may explain the observed discrepancies. However, significant overlap was observed between genes mis-regulated in *ett-3* mutants between this study and that of Simonini et al. (2017) (Fig. S2; Tables S2 and S3).

**Fig. 1.**
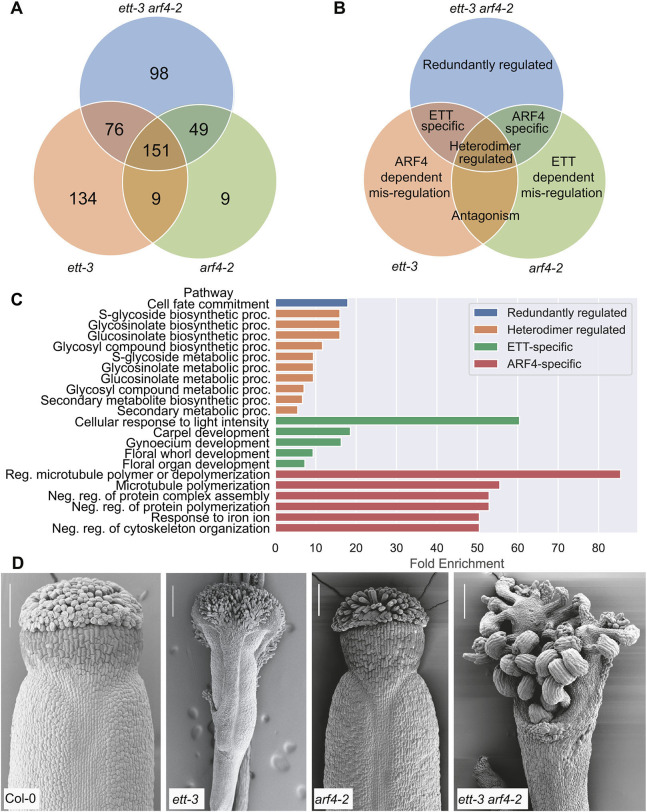
**Summary of genes identified in comparative RNA-seq experiments as ETT/ARF4 targets regulated independently of auxin.** (A) The numbers of genes falling within each segment of a three-way Venn diagram comparing genes that are significantly differentially expressed independently of auxin in *ett-3*, *arf4-2* and *ett-3 arf4-2* mutants compared to wild type. (B) Summary of the relationship between ETT, ARF4 and the genes within each Venn diagram segment. (C) Significant GO terms identified for lists of genes in each Venn diagram segment. (D) The gynoecium phenotypes observed in the lines used for comparative RNA-seq analysis. Scale bars: 100 µm.

The data were analysed to identify auxin-independent ETT/ARF4 targets in accordance with the schema in Fig. S3. Two datasets each composed of three lists of genes were used for this analysis. The first dataset comprised lists of genes that were differentially expressed relative to wild type in *ett-3*, *arf4-2* and *ett-3 arf4-2* after mock treatment, whereas the second dataset comprised the same after IAA treatment. These lists were compared to generate two three-way Venn diagrams, one for each dataset. The data were then compared across mock and auxin treatments to identify genes that are mis-regulated in the same manner in both the presence and absence of auxin. Any auxin-sensitive genes within these lists were removed, leaving genes that are regulated by ETT and ARF4 independently of auxin. Based on where these genes are positioned within a three-way Venn diagram, models of gene behaviour were devised that may explain the observed patterns of statistical significance (Fig. 1; Fig. S4). The gynoecium phenotypes of the lines used in the RNA-seq analysis are shown in Fig. 1D.

Gene Ontology (GO) term analyses were conducted in Shiny GO (Version 0.77, Ge et al., 2020) to identify biological processes that are enriched within each target category (Fig. 1C) compared to a background list composed of all genes expressed in the RNA-seq experiment.

### ETT and ARF4 redundantly regulate cell fate commitment independently of auxin in *Arabidopsis*

**Fig. 2.**
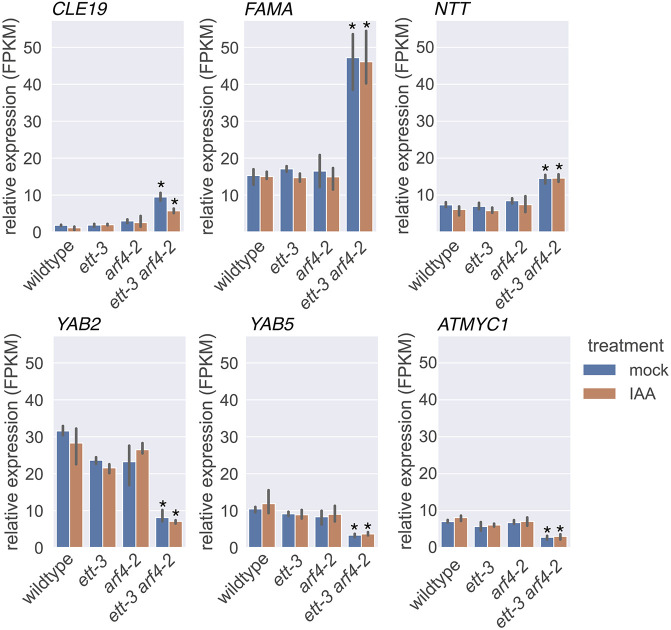
**The normalized expression of genes that are redundantly regulated by ETT and ARF4 independently of auxin, and were identified by GO term analysis as genes involved in the regulation of cell fate commitment.** *CLE19*, *CLAVATA3/ESR-RELATED 19*; *NTT*, *NO TRANSMITTING TRACT*; *YAB2*, *YABBY2*; *YAB5*, *YABBY5*; *ATMYC1*, *ARABIDOPSIS THALIANA MYC1*; FPMK, fragments per kilobase per million mapped fragments. Statistical analysis was conducted using read count normalization, negative binomial distribution analysis and FDR correction using the BH procedure. Data are mean±s.d. *P adjust<0.05 versus the same treatment in wild type.

The transcriptomics analysis identified 98 genes (53 upregulated, 45 downregulated) that were significantly mis-regulated in *ett-3 arf4-2* relative to wild type in accordance with their redundant regulation by ETT and ARF4 independently of auxin (Fig. 1A,B; Fig. S4). GO term analysis indicated significant enrichment within this list for genes involved in ‘cell fate commitment’ (Table 1; Fig. 1C), including *CLAVATA3/ESR-RELATED 19* (*CLE19*), *NO TRANSMITTING TRACT* (*NTT*) and *FAMA*, which were significantly upregulated; and *YABBY2* (*YAB2*) and *YAB5*, and *ATMYC1*, which were significantly downregulated in *ett-3 arf4-2* (Fig. 2; Table 1).

**Table 1.** Genes that are significantly differentially expressed in *ett-3 arf4-2* mutants relative to wild type both in the presence and absence of auxin, consistent with their redundant regulation by ETT and ARF4

Locus	Name	Log2Fold versus wild-type mock in *ett-3 arf4-2* mock	Log2Fold versus wild-type IAA in *ett-3 arf4-2* IAA	Significant in all treatments	GO term
AT3G24225	*CLE19*	2.39	2.27	True	Cell fate commitment
AT3G57670	*NTT*	1.01	1.22	True	Cell fate commitment
AT2G26580	*YAB5*	−1.6	−1.7	True	Cell fate commitment
AT3G24140	*FAMA*	1.66	1.57	True	Cell fate commitment
AT1G08465	*YAB2*	−1.94	−2.04	True	Cell fate commitment
AT4G00480	*ATMYC1*	−1.29	−1.46	True	Cell fate commitment
AT1G70560	*TAA1*	1.61	1.34	True	Indole-acetic acid biosynthetic process
AT5G03790	*ATHB-51*	1.15	1.13	True	Floral meristem determinacy
AT2G33880	*WOX9*	1.66	1.90	True	Regulation of meristem growth
AT1G80080	*TMM*	−1.04	−1.09	True	Stomatal complex formation

The YABBY transcription factors *YAB2* and *YAB5*, which are implicated in abaxial cell fate specification, were constitutively downregulated in the double mutant (Table 1; Fig. 2). *FAMA*, a regulator of guard cell differentiation in the stomatal lineage, was upregulated in *ett-3 arf4-2*, and *TOO MANY MOUTHS* (*TMM*), another stomatal regulator, was constitutively downregulated (Table 1). Along with the mis-regulation of stomatal lineage genes in *ett-3 arf4-2*, stomatal defects were also observed on the abaxial surface of *ett-3 arf4-2* leaves (Fig. S5). Finger-like protrusions, which resemble the midvein in wild-type plants, were produced from the abaxial surface of *ett-3 arf4-2* leaves (Fig. S5B,C). In wild type, the midvein lacks stomata, but the projections in *ett-3 arf4-2* exhibited stomata across the surface of these structures (Fig. S5C). The *ett-3 arf4-2* mutant also shows morphological defects in its gynoecia that fail to produce valves and exhibit ectopic ovules (Fig. 1D).

The WUSCHEL-related homeobox transcription factor *WOX9*, the CLAVATA3-related peptide encoding gene *CLE19* and the gene encoding HD-ZIP I transcription factor *ARABIDOPSIS THALIANA HOMEOBOX 51* (*ATHB-51*) were constitutively upregulated in *ett-3 arf4-2* (Table 1); these genes are implicated in meristematic regulation. Consistent with a role for ETT/ARF4 in the regulation of meristem identity, variability in the numbers of floral organs produced within *ett-3 arf4-2* flowers was observed (Fig. S5D-H), suggesting a role for ETT and ARF4 in floral meristem regulation. The auxin biosynthesis gene *TAA1*, which promotes the production of auxin through the IPA pathway, was constitutively upregulated in the double mutant (Table 1), suggesting that auxin biosynthesis is also regulated redundantly by ETT and ARF4 independently of auxin. Together, these data suggest that ETT and ARF4 may redundantly regulate cell fate specification independently of auxin in a range of biological contexts.

### Transcriptomics identify ETT and ARF4-specific targets in *Arabidopsis*

**Fig. 3.**
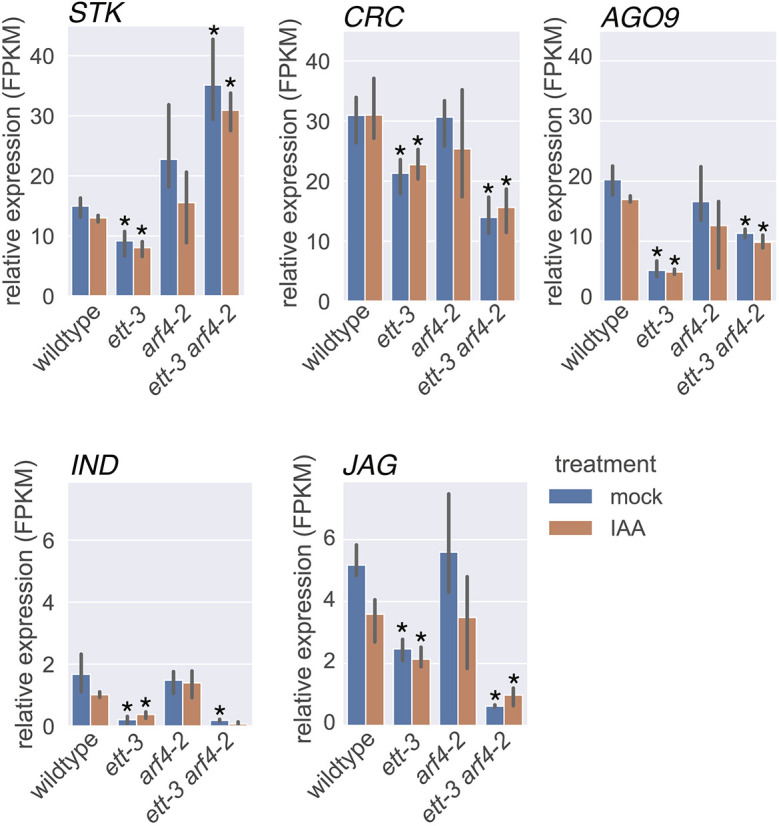
**The normalized expression of genes identified by GO term analysis as involved in gynoecium development that are differentially expressed in *ett-3* and *ett-3 arf4-2* relative to wild type, consistent with their ETT-specific regulation.** *STK*, *SEEDSTICK*; *CRC*, *CRABSCLAW*; *AGO9*, *ARGONAUT9*; *IND*, *INDEHISCENT*; *JAG*, *JAGGED*; FPMK, fragments per kilobase per million mapped fragments. Statistical analysis was conducted using read count normalization, negative binomial distribution analysis and FDR correction using the BH procedure. Data are mean±s.d. *P adjust<0.05 versus the same treatment in wild type.

Transcriptomics revealed 76 targets that became similarly mis-regulated in both *ett-3* and *ett-3 arf4-2*, consistent with their ETT-specific regulation (Fig. 1A,B; Fig. S4). The list of ETT-specific targets was enriched in GO term analysis for genes involved in ‘gynoecium development’, ‘carpel development’, ‘floral whorl development’, ‘floral organ development’ and ‘cellular response to light intensity’ (Table 2; Fig. 1C). The list included the zinc-finger transcription factor *JAGGED* (*JAG*), the YABBY transcription factor *CRABSCLAW* (*CRC*) and basic-Helix-Loop-Helix TF *INDEHISCENT* (*IND*), all of which were constitutively downregulated in *ett-3* and *ett-3 arf4-2* mutants (Table 2; Fig. 3). JAG functions with NUBBIN (NUB) to regulate gynoecium development (Dinneny et al., 2006), and knockdown of *CRC* has previously been shown to cause apical gynoecium defects in *Arabidopsis* (Bowman and Smyth, 1999), while IND regulates valve margin specification (Liljegren et al., 2004). Together, these data suggest that ETT regulates aspects of gynoecium development independently of auxin.

**Table 2.** Genes that are significantly differentially expressed in *ett-3* and *ett-3 arf4-2* mutants relative to wild type both in the presence and absence of auxin, consistent with their ETT-specific regulation

Locus	Name	Col-0 versus *ett-3* mock log2fold change	Col-0 versus ett-3 IAA log2fold change	Col-0 versus *ett-3 arf4-2* mock log2fold change	Col-0 versus *ett-3 arf4-2* IAA log2fold change	Significance in all groups	GO terms
AT1G69180	*CRC*	−0.44	−0.42	−1.12	−1.02	True	Carpel development
AT5G21150	*AGO9*	−1.87	−1.78	−0.82	−0.83	True	Floral organ development
AT4G09960	*STK*	−0.60	−0.68	1.27	1.21	True	Carpel development
AT3G22840	*ELIP1*	1.63	1.75	1.41	1.35	True	Response to light intensity
AT1G29440	*SAUR63*	−1.18	−1.43	−1.08	−1.15	True	Floral organ development
AT1G68480	*JAG*	−0.96	−0.72	−2.97	−1.88	True	Carpel development
AT4G14690	*ELIP2*	1.54	1.13	1.56	1.14	True	Response to light intensity
AT4G00120	*IND*	−2.82	−1.38	−3.03	−3.61	True	No GO term available; valve margin specification (Liljegren et al., 2004)

Forty-nine ARF4-specific genes were identified (Fig. 1A,B; Fig. S4). This list was enriched for genes involved in the ‘regulation of microtubule polymerization or depolymerization’ and related GO terms (Fig. 1C) and did not contain any known regulators of gynoecium development, suggesting that ARF4 mainly functions in conjunction with ETT to elicit its auxin-independent effects in the carpels. Together, the transcriptomics data suggest that ETT and ARF4 regulate distinct and overlapping targets independently of auxin to promote gynoecium development, and imply that ARF4 mainly functions with ETT to promote its auxin-independent effects on gynoecium development.

### Auxin-sensitive analysis in *Arabidopsis* suggests redundant maintenance of auxin insensitivity by ETT and ARF4

Gynoecium morphogenesis is dependent on the dynamic distribution of auxin (Moubayidin and Østergaard, 2014), and gynoecium defects of *ett-3 arf4-2* mutants suggest a role for ETT and ARF4 in the transduction of auxin signalling (Pekker et al., 2005). A second analysis was therefore conducted in *Arabidopsis* to assess how the auxin response varies from wild type in the mutants. Genes that were significantly auxin responsive in wild type were compared to genes that were significantly auxin responsive in each mutant background (*ett-3*, *arf4-2* and *ett-3 arf4-2*).

**Fig. 4.**
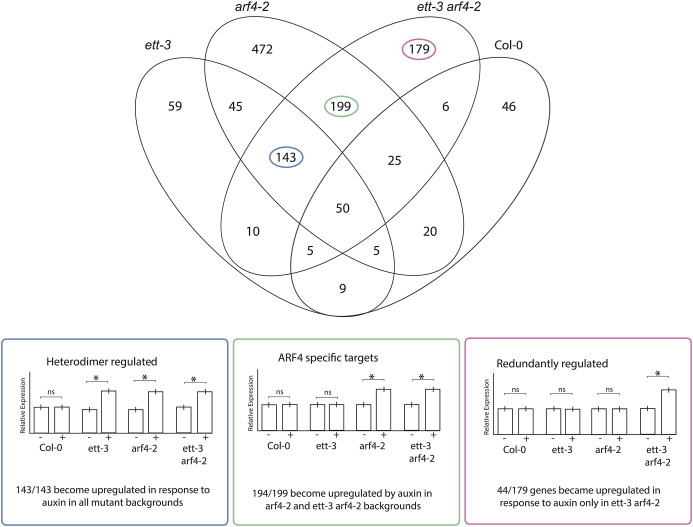
**Summary of the outcome of the auxin-sensitive comparative RNA-sequencing analysis comparing the auxin response in *ett-3*, *arf4-2* or *ett-3 arf4-2* to the auxin response in wild type.** Highlighted on the Venn diagram are groups of genes that conform to models of A-B ARF antagonism (heterodimer regulated are shown in blue, ARF4 specific in green, and redundantly regulated in red). The panels below show the predicted behaviours of genes that conform to these models, and how many genes within each Venn diagram segment conformed to this mode of gene regulation. Asterisks indicate the predicted pattern of significant differential gene expression for loci within each Venn diagram segment. The error bars indicate the s.d. of predicted gene expression patterns.

Few genes were identified in the auxin-sensitive analysis that had been previously implicated in gynoecium development. 179 genes were auxin sensitive only in the *ett-3 arf4-2* mutant background (44 upregulated and 135 downregulated upon auxin treatment, Fig. 4), consistent with their redundant regulation by ETT and ARF4, which maintain their auxin insensitivity. This list was enriched for a range of GO terms, including ‘pectin metabolic process’ and ‘pectin catabolic process’ (Table S8). The mis-regulation of pectin methylesterification has previously been linked to gynoecium defects in *ett* mutants (Andres-Robin et al., 2018), and several pectin methlyesterases (PMEs) and pectin methylesterase inhibitors (PMEIs) have been previously identified as direct ETT targets (Andres-Robin et al., 2020). The list of redundantly regulated ETT/ARF4 targets in the auxin-sensitive analysis conducted here included *PME23*, *PME28*, *PME48* and *PME49*, and members of the pectin lyase superfamily (*AT1G05660*, *AT3G07850*, *AT5G48140*, *AT3G07830*, *AT3G07840* and *AT3G14040*).

None of the other gene lists assessed in the auxin-sensitive analysis contained known direct regulators of gynoecium development, but the data did implicate ETT and ARF4 in the maintenance of auxin insensitivity at target loci involved in a range of biological processes, which then become auxin sensitive upon ETT/ARF4 mutation, consistent with previously proposed models of A-B ARF antagonism.

### Auxin-sensitive analysis reveals patterns of gene regulation consistent with A-B ARF antagonism

**Fig. 5.**
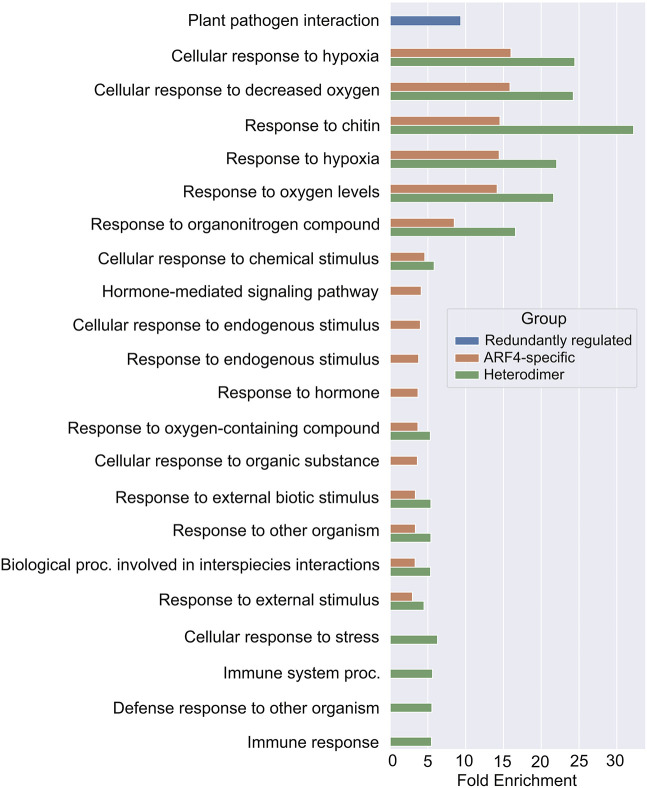
GO terms that are significantly enriched in lists of genes that conform to models of A-B ARF antagonism.

Previous studies in *Marchantia polymorpha* and *Phiscomitrium patens* found that antagonism between A- and B-ARFs regulates the auxin sensitivity of tissues (Kato et al., 2020; Lavy et al., 2016), with the auxin response being promoted by A-ARFs and inhibited by B-ARFs. Within the auxin-sensitive analysis, several groups of genes were identified that behave in a manner consistent with A-B ARF antagonism (Fig. 4). First, 44 genes (out of 179) became upregulated in response to auxin only in *ett-3 arf4-2* mutants, suggesting that ETT and ARF4 act redundantly to prevent their auxin responsiveness (Fig. 4). This list was enriched for genes involved in ‘plant-pathogen interaction’ (Fig. 5). Second, 143 genes (out of 143) were auxin insensitive in wild type and upregulated in response to auxin in all mutant backgrounds, suggesting that ETT-ARF4 dimers maintain auxin insensitivity at these loci (Fig. 4). Interestingly, ETT and ARF4 showed physical interactions in Yeast 2-Hybrid (Y2H) assays (Fig. S6). The ETT-ARF4 interaction was dependent on the presence of the ARF4 DNA-binding domain and was not sensitive to auxin (Fig. S6). Together, the Y2H and RNA seq data suggest that ETT and ARF4 can form heterodimers to regulate gene expression*.*

Finally, 194 genes (out of 199) were auxin insensitive in wild type and became upregulated upon auxin treatment in *arf4-2* and *ett-3 arf4-2* mutants, consistent with the ARF4-specific prevention of their auxin sensitivity (Fig. 4). The list of heterodimer targets that became auxin responsive in all mutant backgrounds was enriched for loci involved in responses to environmental stresses, including ‘response to external biotic stimulus’ and ‘response to hypoxia’ (Fig. 5). The list of ARF4-specific targets was also enriched for similar GO terms (Fig. 5). Together, these data suggest a role for ETT and ARF4 in the regulation of a range of responses to biotic and abiotic stress.

### Synergistic activities of canonical and ETT-mediated signalling components promote gynoecium development in *Arabidopsis*

Previous studies in *Arabidopsis* have hypothesized that ETT and ARF4 function with other ARFs to promote the abaxial identity of lateral organs in response to auxin gradients (Pekker et al., 2005). ETT and ARF4 are B-class ARFs, which are generally regarded as repressors and may therefore antagonize A-class ARF activators to fine-tune auxin signalling, as has previously been shown in *Marchantia* and *Phiscomitrium* (Kato et al., 2020; Lavy et al., 2016).

In line with this hypothesis, ETT represses genes in the absence of auxin by interacting with TPL (Kuhn et al., 2020). In the presence of auxin, ETT undergoes a conformational change and the interaction with TPL is broken, and ETT targets are subsequently derepressed, which is consistent with A-B ARF antagonism at these loci. ETT/ARF4 are broadly co-expressed in the gynoecium with A-Class ARFs, including ARF5, 6 and 8 (Fig. S7). However, the RNA-seq dataset presented here did not identify genes that became upregulated in response to auxin in the mutant backgrounds (consistent with A-B ARF antagonism) with a known role in gynoecium development, making the relationship between the canonical and ETT-mediated pathways in this process unclear.

Previous studies have implicated a range of canonical pathway components in gynoecium development (Dharmasiri et al., 2005; Cecchetti et al., 2008; Prigge et al., 2020; Nagpal et al., 2005). We therefore hypothesized that the canonical pathway may function synergistically to the ETT-mediated pathway in gynoecium development – a relationship that would not be detected in the RNA-seq analysis conducted if canonical and ETT-mediated signalling components are synergistically promoting the expression of shared target genes.

**Fig. 6.**
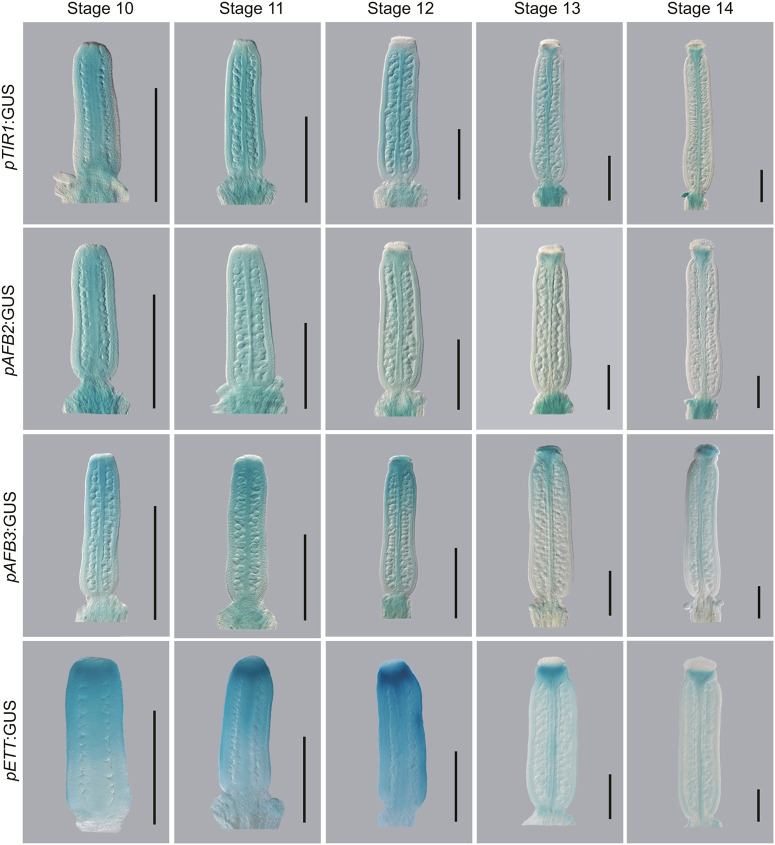
**Expression analysis of genes involved in the canonical and ETT-mediated auxin signalling pathways in *Arabidopsis thaliana.*** GUS staining of gynoecia showing the expression patterns of *AtTIR1, AtAFB2, AtAFB3* and *AtETT* from stage 10 to stage 14 of gynoecium development. Scale bars: 500 µm.

To assess this hypothesis, we first studied the expression of GUS reporter lines for *ETT*, *TIR1*, *AFB2* and *AFB3* during gynoecium development, showing where these genes are expressed under their native promoters (Fig. 6). In *Arabidopsis*, *ETT* is expressed throughout the valves of the gynoecium, and is most highly expressed at the apex of the valves in the region that gives rise to the style at stage 10-12 (Fig. 6). At later stages, *ETT* expression is much lower in the valves and becomes mainly restricted to the style and replum. *AtTIR1*, *AtAFB2* and *AtAFB3* are also expressed throughout the valves during stage 10-11 of gynoecium development, like *AtETT* (Fig. 6). *AtTIR1* expression becomes reduced in the valves after stage 13 of development and is restricted to the style and replum at stage 14. *AtAFB2* is also restricted to the style from stage 13 of gynoecium development, while *AtAFB3* expression remains strongest in the apical valves, stigma and style at stage 12-13 (Fig. 6). *AtAFB3* is then restricted to the style and stigma from stage 14 of gynoecium development.

**Fig. 7.**
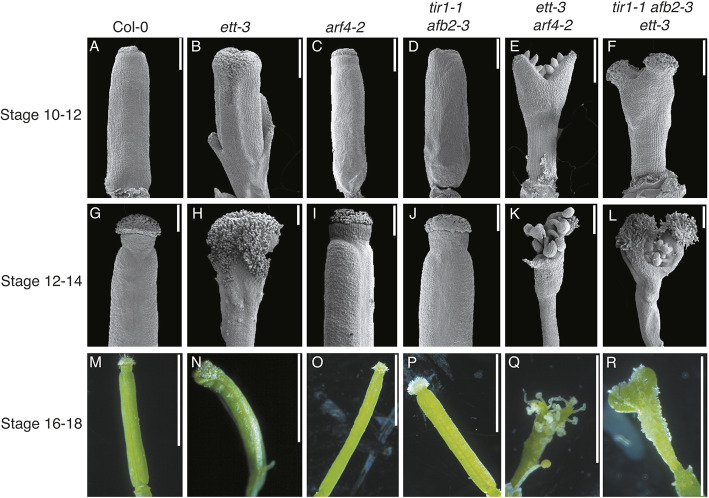
**Phenotypic analysis of *Arabidopsis* gynoecium defects in canonical and ETT-mediated pathway mutants reveals synergism in gynoecium development.** Scanning electron micrographs with backgrounds removed (stage 10-12, stage 12-14), and photographs (stage 16-18, with background cleanup) of gynoecia from wild type (A,G,M), and *ett-3* (B,H,N), *arf4-2* (C,I,O), *tir1-1 afb2-3* (D,J,P), *ett-3 arf4-2* (E,K,Q) and *tir1-1 afb2-3 ett-3* (F,L,R) mutants in *Arabidopsis*. Scale bars: 200 µm in A-L; 0.2 cm in M-R. Original unedited images are displayed in Fig. S10.

The expression of the canonical and ETT-mediated signalling machinery within the same broad domains suggests that both pathways may be involved in gynoecium development. To understand the relationship between the canonical and ETT-mediated pathways during gynoecium development, *tir1 afb2 ett-3* triple mutants were generated in *Arabidopsis*, and the resulting phenotypes were assessed and compared to those produced upon ETT and ARF4 mutation (Fig. 7).

The *ett-3* mutant used in this study exhibits a recessive gynoecium phenotype of intermediate strength (Sessions et al., 1997), which is the product of a nonsense mutation inducing an early stop codon in the ETT protein prior to its C-terminal auxin binding and TPL-interacting domain (Fig. S8). ETT-mediated signalling is thereby abolished in this mutant (Simonini et al., 2016; Kuhn et al., 2020).

The *ett-3* mutant (Fig. 7B,H,N) exhibited reduced valves, a loss of radial symmetry in the style and over-proliferation of stigmatic tissues at later developmental stages (Fig. 7H,N), while the *arf4-*2 (Fig. 7C,I,O) and *tir1-2 afb2-3* (Fig. 7D,J,P) mutants resembled wild type (Fig. 7A,G,M). The *ett-3* phenotype (Fig. 7B,H,N) was exacerbated in both *ett-3 arf4-2* (Fig. 7E,K,Q) and *tir1-1 afb2-3 ett-3* (Fig. 7F,L,R). Valves in *ett-3 arf4-2* gynoecia were significantly reduced, producing an open ovary with ectopic ovules and few stigmatic papillae (Fig. 7E,K,Q). The *ett-3 arf4-2* gynoecia lacked a style and split into two distinct lobes with stigmatic papillae at their tips (Fig. 7E,K,Q). Similarly, *tir1-1 afb2-3 ett-3* mutants exhibited enhanced valve reduction relative to *ett-3* mutants, producing a partially open ovary with ectopic ovules, and no discernable style or replum (Fig. 7F,L,R). The stigmatic papillae in *tir1-1 afb2-3 ett-3* gynoecia also developed atop two lobes produced at the apex, and exhibited overproliferation of stigmatic tissues at later developmental stages comparable to those observed in *ett-3* (Fig. 7B,F,H,L,N,R).

The exacerbation of the *ett-3* phenotype by *tir1-1 afb2-3* mutation demonstrates that the canonical and ETT-mediated pathways function synergistically to promote gynoecium development. The phenotypic similarities between *ett-3 arf4-2* and *tir1-1 afb2-3 ett-3* mutants, especially at the early stages of gynoecium development, suggest that ETT/ARF4 and TIR1/AFB2 regulate gynoecium development along the apical basal axis, and further implicate ARF4 in the integration of signalling with the canonical pathway.

### Conserved auxin signalling synergism in heart-shaped fruit development

**Fig. 8.**
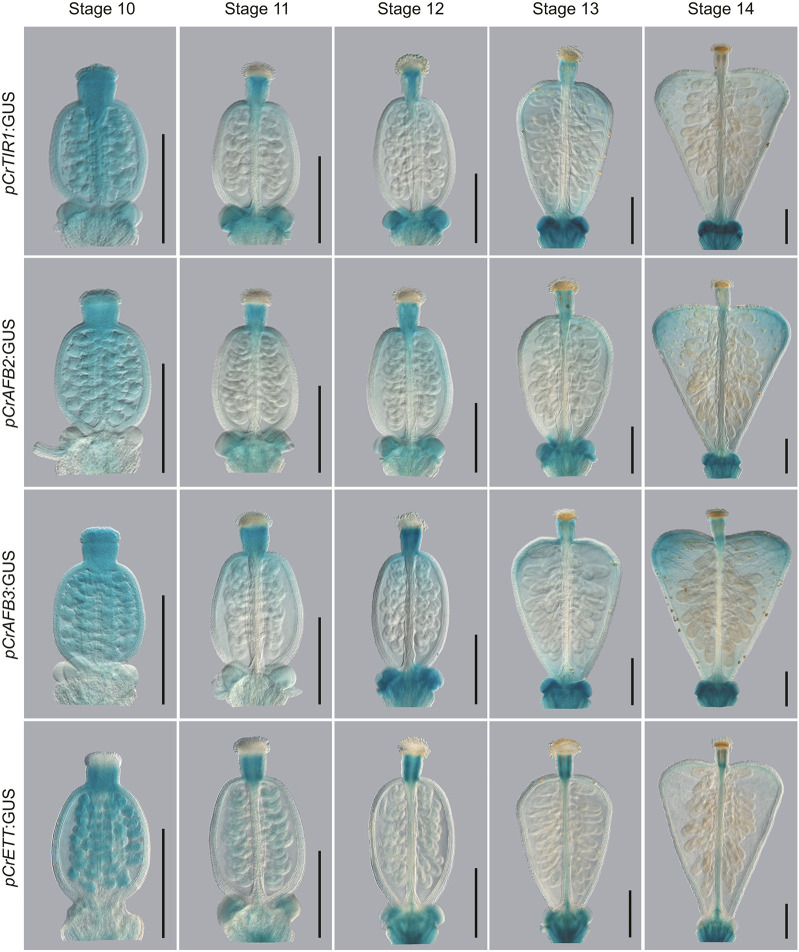
**Expression analysis of genes involved in the canonical and ETT-mediated auxin signalling pathway in *Capsella rubella*.** GUS staining showing the expression patterns of *CrTIR1*, *CrAFB2*, *CrAFB3* and *CrETT* from stage 10 to stage 14 of gynoecium development. Scale bars: 500 µm.

The relationship between canonical and ETT-mediated auxin signalling components has not yet been studied beyond *Arabidopsis*, so it is unclear whether it is relevant to fruit development beyond this model system. To assess whether the synergism between the canonical and ETT-mediated signalling components identified in *Arabidopsis* are also conserved in species with distinct fruit shapes, GUS reporter lines were generated to assess the expression patterns of *ETT*, *TIR1*, *AFB2* and *AFB3* in *Capsella rubella*, a species that diverged from *Arabidopsis* ∼8 million years ago and produces heart-shaped fruits (Fig. 8).

*CrETT* is expressed strongly in the apical tissues of the gynoecium and the ovules during stage 10, while *CrTIR1*, *CrAFB2* and *CrAFB3* are expressed ubiquitously across the gynoecium at this stage (Fig. 8). *CrETT* is then most strongly expressed in the style from stage 11 to stage 14. *CrTIR1*, *CrAFB2* and *CrAFB3* are continually expressed in the apical valves and style from stage 11 to stage 13, and at stage 14 their expression levels are highest in the outgrowing shoulders of the *Capsella* fruit, although *CrTIR1* is expressed at lower levels than *CrAFB2* and *CrAFB3* (Fig. 8). Canonical and ETT-mediated signalling machineries are therefore expressed within broadly similar domains in *Capsella*.

**Fig. 9.**
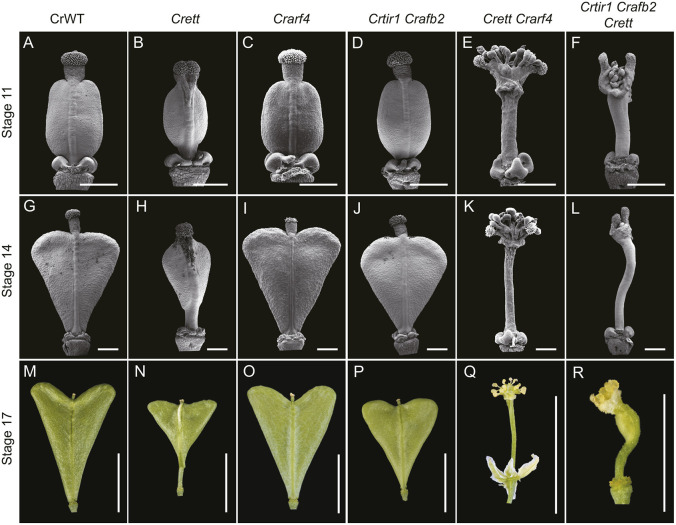
**Phenotypic analysis of *Capsella* gynoecium defects in canonical and ETT-mediated pathway mutants reveals conserved synergism in gynoecium development.** Scanning electron micrographs with backgrounds removed (stage 11 and stage 14), and photographs (stage 17) of gynoecia from wild type (A,G,M), and *Crett* (B,H,N), *Crarf4* (C,I,O), *Crtir1 Crafb2* (D,J,P), *Crett Crarf4* (E,K,Q) and *Crtir1 Crafb2 Crett* (F,L,R) mutants in *Capsella,* Scale bars: 200 µm in A-L; 0.5 cm in M-R. Original unedited images are displayed in Fig. S11.

Next, phenotypic analyses were conducted on the same mutant combinations previously assessed in *Arabidopsis* (*ett*, *arf4*, *ett arf4*, *tir1 afb2* and *tir1 afb2 ett*), which were generated by CRISPR/Cas9 in *Capsella* (Fig. 9). As in *Arabidopsis*, the *Crarf4* mutants (Fig. 9C,I,O) resembled wild type (Fig. 9A,G,M), while *Crett* displayed valve reduction, stigmatic over proliferation and breakage in the radial symmetry of the style (Fig. 9B,H,N). Whereas the *Arabidopsis tir1-1 afb2-3* mutant phenocopied wild type, valve reduction was observed in the *Crtir1 Crafb2* mutant, and this persisted at later developmental stages so that the shoulders of the heart never fully elongated (Fig. 9D,J,P). This suggests a more prominent role for the canonical pathway in valve outgrowth in *Capsella* than in *Arabidopsis* and implicates the canonical pathway in heart-shaped fruit development.

In *Arabidopsis*, the *ett-3 arf4-2* mutant exhibited the strongest phenotypes, producing a completely open ovary, whilst *the tir1-1 afb2-3 ett-3* mutants exhibited less severe valve reduction defects (Fig. 7). However, *Crett Crarf4* (Fig. 9E,K,Q) and *Crtir1 Crafb2 Crett* (Fig. 9F,L,R) mutants exhibited phenotypes that were similar in severity. Both lines produced open ovaries with ectopic ovules atop a hyperextended stalk-like structures (Fig. 9E,F,K,L,Q,R). As in *Arabidopsis*, stigmatic tissues formed at the apex of structures at the lateral edges of the gynoecium (Fig. 7E,F,K,L). Taken together, these data suggest that the synergism between canonical and ETT-mediated pathways in gynoecium development is conserved between *Arabidopsis* and *Capsella*, and may therefore be broadly relevant for gynoecium development across the Brassicaceae and in species with distinct fruit shapes.

## DISCUSSION

### Comparative transcriptomics correlate with phenotypic defects observed in *Arabidopsis* mutants

The auxin-sensitive analysis identified distinct and overlapping targets of ETT and ARF4, and found that these ARFs have both auxin-dependent and -independent targets. Targets under the auxin-independent redundant regulation by ETT and ARF4 were enriched for genes involved in cell fate specification and included the YABBY TFs *YAB2* and *YAB5. CRC*, another *YABBY* TF, was mis-regulated in both *ett-3* and *ett-3 arf4*-2, consistent with its ETT-specific regulation, and has a known role in gynoecium development (Bowman, 2000). While *yabby* single mutants lack obvious abaxial-adaxial identity defects, higher-order mutants show significant alterations in abaxial-adaxial polarity (Sarojam et al., 2010; Siegfried et al., 1999).

YABBY proteins specify abaxial identity along with the KANADI family of transcription factors (Eshed et al., 2004). ETT and ARF4 function co-operatively with KANADI proteins to promote abaxial cell fate of lateral organs (Pekker et al., 2005). Laminal outgrowths from the abaxial leaf surface have previously been observed in *kan1 kan2* loss-of-function mutants (Eshed et al., 2004), *ett arf4 arf2* triple mutants (Guan et al., 2017), and the *ett-3 arf4-2* mutant leaves in this study and previous studies (Pekker et al., 2005), consistent with previously proposed roles for ETT/ARF4 in KANADI-mediated signalling.

Previous studies reported reduced valve margin definition in *ind* (Liljegren et al., 2004), and apical gynoecium defects in *jag nub* and *crc* mutants (Dinneny et al., 2006; Bowman and Smyth, 1999). Similar gynoecium defects were exhibited in this study by *ett-3* and *ett-3 arf4-2* (Fig. 1), along with the constitutive downregulation of *IND*, *JAG* and *CRC*, which is consistent with the previously proposed roles of these genes in gynoecium development. Future studies should aim to determine which of the targets identified within this study are direct/indirect ETT/ARF4 targets, and to validate the roles of the targets identified in the regulation of gynoecium development in both *Arabidopsis* and *Capsella*.

### Auxin-sensitive analysis suggests ETT and ARF4 maintain auxin insensitivity at target loci

Previous studies in *Marchantia polymorpha* and *Phiscomitrium patens* found antagonism between A- and B-ARFs to regulate the auxin sensitivity of tissues (Kato et al., 2020; Lavy et al., 2016), with the auxin response being promoted by A-ARFs and inhibited by B-ARFs. Consistent with this model of B-ARF function, ETT and ARF4 were previously shown to repress auxin responses in an *Arabidopsis* root hair system (Choi et al., 2018). The transcriptomics data presented here suggest that ETT and ARF4 largely prevent the auxin sensitivity of distinct and overlapping targets, many of which become upregulated in response to auxin in various mutant backgrounds, consistent with models of A/B-ARF antagonism.

However, many of their redundantly regulated targets, including genes involved in the regulation of pectin, become downregulated in response to auxin. Auxin-mediated repression has been observed in TMK-mediated signalling, where atypical AUX/IAAs are stabilized in the presence of auxin (Cao et al., 2019). However, it is unclear if such a mechanism functions in the gynoecium. Previous studies have implicated PME family members in the regulation of cell wall stiffness during gynoecium development in *ett* mutants and show that ETT promotes PME expression in the carpels (Andres-Robin et al., 2018, 2020). Furthermore, the overexpression of *PECTIN METHYLESTERASE INHIBITOR 3* (*PMEI3*), which antagonizes PME activity, phenocopied the valve reductions observed in *ett* mutants (Andres-Robin et al., 2018). Taken together with these previous studies, the data presented here suggest that ETT and ARF4 may redundantly promote the expression of PMEs to prevent their downregulation in response to auxin, and the mis-regulation of these genes may contribute to the gynoecium defects observed in *ett-3 arf4-2*.

The data presented suggest that ETT functions alongside ARF4 to prevent the expression of genes that would otherwise become auxin sensitive in its absence, possibly through the activity of A-ARFs with which they are co-expressed or due to the activity of TMK-related pathways, in the case of auxin-downregulated genes. The data also suggest that the maintenance of genes in an auxin-insensitive state may be equally as important as the promotion of auxin sensitivity at other loci to facilitate normal growth and development. This study demonstrates how dissecting the relationships between distinct auxin signalling pathways is a powerful approach with which to gain a greater understanding of the wider auxin signalling network, thereby broadening the perspective of auxin-mediated plant development.

### Synergism between canonical and ETT-mediated signalling components in gynoecium development

The phenotypic and expression analyses presented here suggest the existence of synergistic regulation between the canonical and ETT-mediated pathways in gynoecium development, and that this mechanism is conserved between *Arabidopsis* and *Capsella*. Indeed, previous studies in both *Arabidopsis* and *Capsella* have shown that the dynamic distribution of auxin plays an important role in gynoecium development (Moubayidin and Østergaard, 2014; Dong et al., 2019).

Whereas a role for ETT in style development has been observed in *Brassica rapa* (Simonini et al., 2018), a role for ETT in *Capsella* fruit development had not been characterized prior to this study. In *Arabidopsis*, ETT was known to function partially redundantly with ARF4 in gynoecium development (Pekker et al., 2005; Finet et al., 2010). Here, we show that, as in *Arabidopsis*, ETT and ARF4 function partially redundantly in *Capsella*. The *Crett* mutant phenotype was exacerbated in *Crett arf4*, suggesting that ARF4 shares some, but not all, of its functionality with ETT.

Furthermore, the *tir1 afb2 ett* triple mutant phenocopies *ett arf4* mutants in both species studied, suggesting a role for ARF4 in the integration of signalling with the canonical pathway. Consistent with this hypothesis, ARF4 has previously been shown to interact with AUX/IAAs of the canonical pathway through its C-terminal domain (Vernoux et al., 2011).

Structural evolution studies have previously indicated that ETT and ARF4 arose from the duplication of a non-truncated ARF prior to the radiation of extant angiosperms (Finet et al., 2010), and that the truncation of either ETT or ARF4, so that they lack the C-terminal PB1 domain, is common in the angiosperm lineage. However, while *Arabidopsis* ETT was more able to rescue *ett* phenotypes when truncated, ARF4 required its C-terminal domain to function (Finet et al., 2010), consistent with the idea that AUX/IAA interaction is required for ARF4 activity. However, the C-terminal PB1 domain has also been shown to facilitate ARF homodimer and homo-oligomerization (Davis et al., 2020; Korasick et al., 2015), meaning that further studies will be required to understand the mechanism of ARF4 action, and how it integrates the canonical and ETT-mediated pathways. It also remains unclear how the ETT/ARF4 activity observed relates to the recruitment of TPL, and regulation of the chromatin landscape, which would be an interesting area for future study.

### Auxin signalling-mediated patterning of lateral organs

Previous studies had posited firstly that activating and repressive ARFs antagonize one another to regulate levels of gene expression (Kato et al., 2020; Lavy et al., 2016), and secondly that ETT and ARF4 function with other ARFs to translate auxin gradients into tissue polarities in lateral organs (Pekker et al., 2005). With these observations in mind, it was originally hypothesized that ETT-ARF4 targets that regulate gynoecium development would be identified in the auxin-sensitive RNA-seq analysis and would conform to models of A-B ARF antagonism. None of the genes identified in this study that behave in accordance with A-B ARF antagonism were known regulators of gynoecium development. However, previous studies had also implicated canonical pathway signalling components in the regulation of gynoecium development (Dharmasiri et al., 2005; Cecchetti et al., 2008; Prigge et al., 2020; Nagpal et al., 2005), leading to a second hypothesis that the relationship between the canonical and ETT-mediated pathways in gynoecium development may be synergistic. The data presented here support the latter hypothesis.

During gynoecium development, the dynamic distribution of auxin changes, initially producing auxin signalling foci through the transport of auxin from the base to the apex of the gynoecium (Moubayidin and Østergaard, 2014). This is required for valve elongation and appropriate development of the gynoecium along the apical basal axis. Later in development, a ring of auxin forms at the apex of the style, triggering its radialization (Moubayidin and Østergaard, 2014). In both *Arabidopsis* and *Capsella*, *ett*, *ett arf4* and *tir1 afb2 ett* mutants exhibit loss of radialization in the apex of the gynoecium or the loss of a style entirely, and significant valve reduction, suggesting that these pathways may translate the dynamic auxin distribution into appropriate developmental outputs, even in species with distinct fruit shapes.

Future studies should continue to dissect the relationships between distinct auxin signalling pathways in *Arabidopsis* and beyond, both through the generation of novel combinations of higher order mutants and comparative transcriptomics. Together these approaches provide novel insights into the wider auxin signalling network, revealing roles in plant development that can otherwise be masked by genetic redundancy.

## MATERIALS AND METHODS

### Plant materials and growth condition

#### Arabidopsis

Seeds were sown onto damp soil (Levington F2 compost with Intercept and grit at a 6:1 ratio) and stratified for 3 days at 4°C in the dark before transferral to a controlled environment room (CER) and growth under long day conditions (16 h light/8 h dark, 22°C). All mutant lines were generated in a Col-0 background. Mutant and transgenic lines used in this work that have been previously published are: *ett-3* (Sessions et al., 1997; Simonini et al., 2016), *arf4-2* (SALK_070506, Alonso et al., 2003) and *tir1-1 afb2-*3 (Parry et al., 2009). The *ett-3 arf4-2* line was generated by crossing the *ett-3* line and *arf4-2* (SALK_070506). The *tir1-1 afb2-3 ett-3* line was generated by crossing *ett-3* with *tir1-1 afb2-3*. The PCR conditions and genotyping primers used for these lines are listed in Tables S4 and S5, respectively. *pARF3/ETT: GFP*, *pARF4: GFP*, *pARF5: GFP*, *pARF6: GFP* and *pARF8: GFP* have been previously published (Rademacher et al., 2011). The *pETT:GUS* line was previously published by Kuhn et al. (2020).

#### Capsella

The genetic and expression analysis in *Capsella rubella* was conducted in the Cr22.5 background. The seeds were germinated on MS medium containing 10 µM Gibberellin at 22°C. Ten-day-old seedlings were then transplanted to soil in 8 cm pots and moved into a controlled environment room (CER) at 22°C, 16 h light and 20°C 8 h dark conditions.

### Genotyping

Genomic DNA was extracted from fresh young leaves by grinding with a micro-pestle in a 1.7 ml tube. *Arabidopsis* extraction buffer was added [200 mM Tris HCl (pH 7.7), 250 mM NaCl, 25 mM EDTA and 0.5% SDS] and the sample was ground for an additional 30 s. The sample was then centrifuged at 15,000 ***g*** in an Eppendorf 5425 table-top centrifuge for 5 min. The supernatant was transferred to a fresh 1.5 ml tube containing 200 µl isopropanol without disturbing the pellet. The sample was then inverted at room temperature for 2 min. The sample then underwent centrifugation at 15,000 ***g*** in a table-top centrifuge for 7 min, and the supernatant was discarded. The pellet was then air dried for 20 min at room temperature and resuspended in 50 µl distilled H_2_O before being used as a template for PCR or frozen at −20°C. SALK lines (*arf4-2 and afb2-3*) were genotyped in accordance with Alsonso et al. (2003), using the primers and conditions specified in Tables S4 and S5. Genotyping PCRs were conducted using GoTaq DNA Polymerase (Promega) in accordance with the manufacturer's guidelines, and with a final volume of 20 µl, where genotype assessment occurred through gel electrophoresis (*arf4-2 and afb2-3*), or of 50 µl if genotype was to be assessed by sequencing (*ett-3*) or BsaI restriction digestion (*tir1-1*). The *ett-3* EMS mutation causes a G to A substitution at position 2226 of the *AT2G33860* genomic sequence. PCR was conducted using the primers specified in Tables S4 and S5, to amplify a 246 bp product that was purified with a QIAquick PCR purification kit (Qiagen) before sequencing. The *tir1-1* allele is an EMS mutation that introduces a G-to-A mutation in position 440 of the genomic *AT3G62980* sequence. A 517 bp product was amplified using the primers specified in Tables S4 and S5, and digested with BsaI-HFv2 (NEB) according to the manufacturer's guidelines before gel electrophoresis of the resulting product on 3% agarose gel. BsaI digestion of the PCR product in the *tir1-1* mutant yields a single band of 517 bp, while the wild type produces two bands of 437 and 80 bp.

### Plasmid construction and plant transformation

For the construction of the promoter, GUS reporter plasmids, the promoter regulatory sequences of *CrAFB2* (1941 bp), *CrAFB3* (2072 bp), *CrTIR1* (2077 bp), *CrETT* (3029 bp), *AtAFB2* (1086 bp), *AtAFB2* (2049 bp) and *AtTIR1* (2076 bp) were isolated by PCR from genomic DNA and inserted upstream of *GUS* gene of pCambia1301 vectors. For the construction of the CRISPR/Cas9-mediated genome editing plasmids, DNA sequences encoding gRNAs adjacent to the PAM sequences (NGG) were designed to target the first or second exons of gene of interest. The gRNAs were synthesized as oligonucleotides with golden-gate cloning adapters and then were insert downstream of U6 promoters. The resulting gRNA plasmids were then recombined with *pRPS5a:Cas9z:E9t* and hygromycin selection marker using golden-gate cloning methods to produce the binary vectors. All vectors were verified by sequencing and introduced into *Agrobacterium tumefaciens* strain LBA4404 by electroporation.

Transformation of *Capsella* plants followed the methods described by Dong et al. (2022). The transformants were screened on MS plants with 1% sucrose and 0.8% agar containing 40 mg/l hygromycin (Roche, 10843555001). For each construct, at least 10 independent transformants were obtained for further analysis. For the CRISPR transformants, preliminary genotyping was conducted using the primers listed in Table S6. Only the plants showing evidence of gene editing were kept and processed into the T2 generation. For generating the Cas9-free homozygous mutant, the T2 CRISPR transformants were screened on MS plants with 1% sucrose and 0.8% agar containing 10 mg/l hygromycin. The hygromycin-sensitive plants were isolated and genotyped again using primers listed in Table S6. All the analysis were conducted on the T2 generation of the representative transgenic lines. Additional information on the mutations induced in each line is available in Fig. S9.

### Expression analysis by GUS staining

The GUS histochemical assays were performed as previously described by Dong et al. (2019). Specifically, inflorescence samples of *Capsella* or *Arabidopsis* were fixed in acetone for 20 min at −80°C, and then washed twice for 5 min in 100 mM sodium phosphate buffer. The samples were then processed in 100 mM sodium phosphate buffer containing 1 mM K_3_Fe(CN) and 1 mM K_4_Fe(CN)_6_ at room temperature for 30 mins to block the diffusion of GUS proteins. The staining process was conducted at 37°C in the X-Gluc solution for 6-8 h. The X-Gluc solution contains 100 mM sodium phosphate buffer, 10 mM EDTA, 0.5 mM K_3_Fe(CN)_6_, 3 mM K_4_Fe(CN)_6_, 0.1% Triton X100 and 1 mg/ml of β-glucoronidase substrate X-gluc (5-bromo-4-chloro-3-indolyl glucuronide, MELFORD: B40020) dissolved in DMSO. After staining, the reaction was suspended and replaced with 70% ethanol several times until chlorophyll was completely washed out. Gynoecia or young fruits were dissected, mounted in chlorohydrate (Sigma number: 15307-500G-R) solution and analysed using a Zeiss Axio Imager light microscope.

### Scanning electron microscopy

The scanning electron microscopy (SEM) experiment followed the protocol of Dong et al. (2019). Specifically, the gynoecia of each genotype were fixed in FAA and dissected. The samples were critical-point dried in CO_2_ and sputter-coated with gold. The samples were subsequently examined using a Zeiss Supra 55VP field scanning electron microscope with an acceleration voltage of 3.0 kV. The SEM images were edited in Photoshop to remove the background. The original unedited images are displayed in Figs S10 and S11.

### RNA-sequencing analysis (*Arabidopsis*)

Whole inflorescences were harvested from Col-0, *ett-3*, *arf4-2* and *ett-3 arf4-2* lines treated with either mock (distilled H_2_O, 0.1% Tween and 0.1% ethanol) or 100 μM IAA and 10 μM NPA (dissolved in ethanol), and 0.1% Tween for 1 h. Three biological replicates were harvested for each genotype 1 h after treatment. Each replicate contained pooled material from three plants. The tissue was snap frozen in liquid nitrogen and RNA extraction was conducted using a Qiagen RNeasy Plant Mini Kit with on-column DNase treatment, in accordance with the manufacturer's guidelines. The RNA quality was assessed by gel electrophoresis; the RNA concentration was measured using a nanodrop (ThermoFisher); and the RNA integrity was tested using a Bioanalyzer 2100 (Agilent). Novogene conducted cDNA library preparation (250-300 bp insert cDNA library) and sequencing (20 M pair reads). The mRNA was enriched using oligo-dT beads and fragmented before cDNA synthesis with reverse transcriptase. First-strand synthesis was conducted and second-strand synthesis buffer (containing dNTPs, RNase H and *E. coli* polymerase I, Illumina) was added so that the second strand was produced by nick translation. The library was then purified and underwent terminal repair, before A-tailing, ligation of sequencing adapters, size selection and PCR enrichment. Library concentration was assessed using a Qubit 2.0 fluorometer (Life Technologies) and the strand sizes were verified using a Bioanalyzer 2100 (Agilent) and qPCR. Raw Illumina sequencing data was then converted to sequence reads using base calling. The error rate (0.03%) and A/T/G/C content distribution were tested as a quality control, before data filtering to remove low quality reads and reads with adaptor contamination. Sequencing reads were mapped to the reference genome (TAIR10), using HISAT2. Gene expression level was assessed using transcript abundance, which assesses the number of fragments per kilobase of transcript sequence per million base pairs sequenced (FPKM). This method accounts for both the effects of sequencing depth and gene length on fragment counting. HTSeq software was utilized in this analysis. Differentially expressed gene (DEG) analyses used DESeq2 to conduct read count normalization and negative binomial distribution analysis (*P*<0.05) followed by the determination of the false discovery rate using the Benjamini and Hochberg procedure (*P*_adjust_<0.05). The FPKMs of all genes expressed in this analysis are available in Table S7.

### Principle component analysis

Principle component analysis was conducted in iDEP 9.1 (http://ge-lab.org/idep/; Ge et al., 2018) on expression values normalized to FPKM.

### Go term analysis

Gene Ontology (GO) term analysis was conducted on the lists of genes that fell into each category of the Venn diagrams in both the auxin-independent and auxin-sensitive analyses to identify whether these lists were enriched for genes involved in specific biological processes. This GO term analysis was conducted using shiny GO (version 0.77, Ge et al., 2020) using the default parameters (FDR cut off =0.05). Gene lists were compared to all genes expressed in the RNA-seq analysis (Table S8).

### Analysis of auxin-independent ETT/ARF4 targets

This analysis was conducted using the differentially expressed gene lists provided by Novogene. Six lists of differentially expressed genes were used in this analysis: (1) *arf4-2* vs Col-0 mock; (2) *ett-3* vs Col-0 mock; (3) *ett-3 arf4-2* vs Col-0 mock; (4) *arf4-2* vs Col-0 IAA; (5) *ett-3* vs Col-0 IAA; and (6) *ett- 3 arf4-2* vs Col-0 IAA. Only genes that were at least twofold up- or downregulated in at least one background were included in this analysis (log2fold value of >1 or<−1). Analyses were conducted with Venny (Version 2.1; https://bioinfogp.cnb.csic.es/tools/venny/index.html; Oliveros, 2007) to identify genes mis-regulated in only one background, mis-regulated in only two backgrounds or mis-regulated in all three backgrounds relative to wild type. The mock and auxin-treated datasets were independently assessed using Venny, and then compared to identify genes that were similarly differentially expressed in both the presence and absence of auxin. Finally, any genes that were significantly differentially expressed (twofold up- or downregulated in at least one background) in response to auxin were removed from the lists, leaving only those genes that were regulated independently of auxin. The output of this analysis is available in Table S9.

### Analysis of auxin-sensitive ETT/ARF4 targets

The auxin sensitive analysis utilized DEGs from the following lists generated by Novogene: (1) Col-0 IAA versus Col-0 mock; (2) *ett-3* IAA versus *ett-3* mock; (3) *arf4-2* IAA versus *arf4-2* mock; and (4) *ett-3 arf4-2* IAA versus *ett-3 arf4-2* mock. The genes that were differentially expressed in each background in response to auxin were then compared to reveal how the auxin response in each mutant differed from wild type. Venny (Version 2.1) was used to generate a four-way Venn diagram. The output of this analysis is available in Table S10.

### Yeast 2 hybrid

The full-length coding sequence of ETT and ARF4 and truncated ARF4 fragments were amplified by PCR using primers designed for gateway cloning (Table S5). The resulting PCR products were purified using a Qiagen QIAquick PCR purification kit and introduced in the gateway entry vector pDONR207 (Life Technologies) by BP clonase reactions, in accordance with the Thermo-Fisher Gateway protocol. The products of this reaction were transformed into DH5α cells (Invitrogen) according to the manufacturer's guidelines and grown on LB agar plates containing gentamycin (10 μg/ml) overnight at 37°C. Constructs were validated by colony PCR and sequencing. The ARF sequences within pDONR207 were then recombined into the pGAD-T7 and pGBK-T7 (Clontech) expression vectors by LR clonase reactions, transformed into *E. coli*, and once more validated by colony PCR and sequencing. The expression vectors were then co-transformed into AH109 *Saccharomyces cerevisiae* (Clontech) by lithium acetate-mediated transformation (Gietz and Schiestl, 2007). Different combinations of AD (pGAD-T7) and BD (pGBK-T7) vectors, or empty vector controls, were introduced in pairs to competent yeast cells to determine whether ARF4 and ETT interact, and through which domains of ARF4. Transformants were plated onto YSD -W-L media and grown at 28°C for 72 h, before serial dilution (10^0^, 10^1^, 10^2^ and 10^3^) and plated onto YSD -W-L-H-A plates to assess interactions. After plating on selective media, yeast colonies were photographed after growth at 28°C for 7 days. Figures were created using images from the 10^1^ dilution in all cases. Auxin sensitivity was assessed by adding 100 μM IAA (Sigma) to the medium.

### Confocal microscopy

Stage 8-14 gynoecia were dissected from the plant and placed onto slides in water containing 0.015% Silwet L-77 (De Sangosse) to prevent the formation of bubbles. Coverslips were then applied, and the gynoecia were imaged using a Leica SP5 confocal microscope with a HyD detector. The following settings were used: Smart Gain 30%, scanning speed 200 Hz and 4× line averaging. The images were obtained using a 40× water immersion lens. GFP was excited at a wavelength of 488 nm and detected at 498-530 nm. Images were taken as *z*-stacks, and then processed in Image J 1.48 to form composite 3D projections, which were then converted to RGB images.

## Supplementary Material



10.1242/develop.204263_sup1 Supplementary information

Table S7. FPKMs of all genes expressed in this study

Table S8. GO term analyses conducted in this study

Table S9. Results of the auxin independent analysis conducted in this study

Table S10. Results of the auxin sensitive analysis conducted in this study
