# Supplementary material for: Conserved roles of ETT and ARF4 in gynoecium development in Brassicaceae with distinct fruit shapes
Source: Development. 2025 Feb 12;152(3):DEV204263. doi: 10.1242/dev.204263 (PMC11883277; doi:10.1242/dev.204263)
Supplement: Supplementary information [file develop-152-204263-s1.pdf]

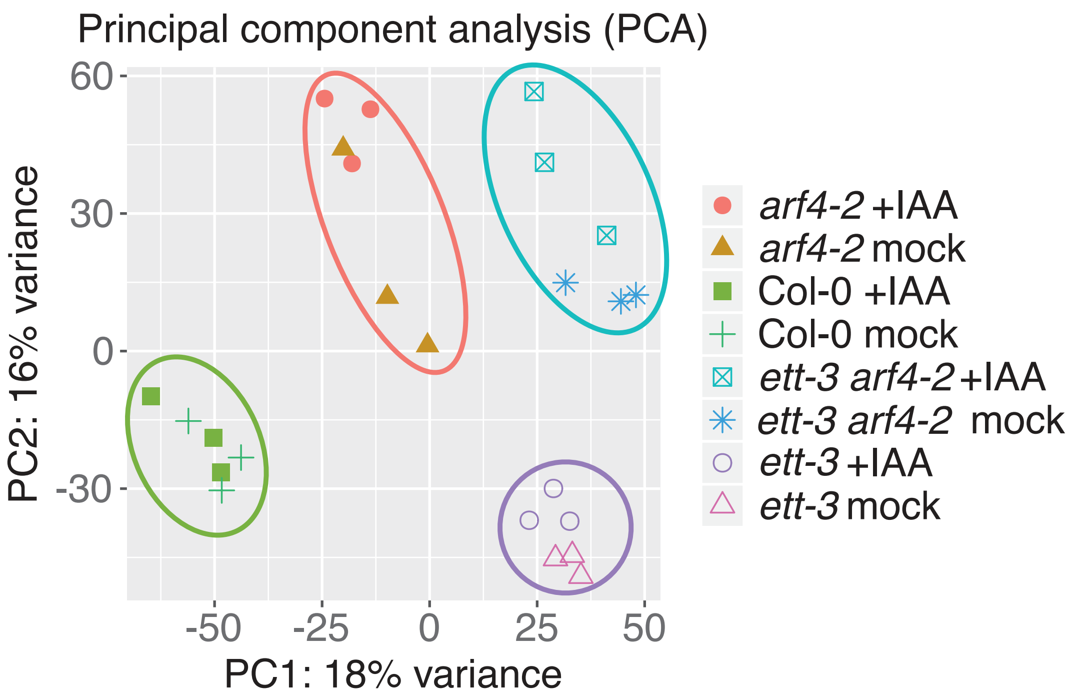

Fig. S1. Principal component analysis showing clustering of data from each line in the comparative RNA seq experiment in *Arabidopsis*.

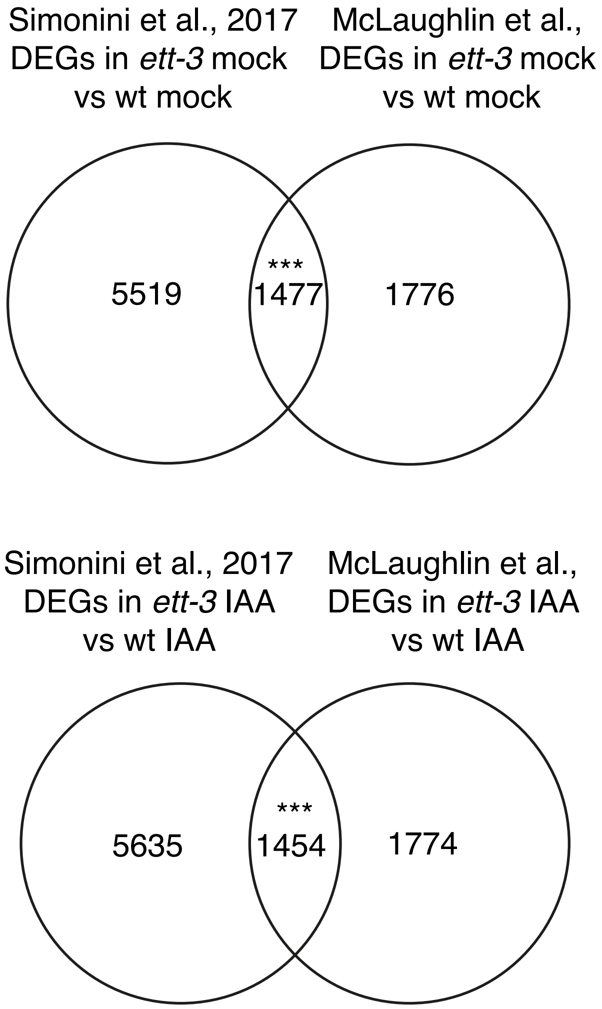

Fig. S2. Venn diagrams summarizing the overlap between the RNA- sequencing data from Simonini et al. 2017 and those presented in this study. DEG, differentially expressed genes. Statistical analysis of overlap was conducted using Fisher's Exact Test $p<0.05$. ***= $p<0.001$

Development • Supplementary information

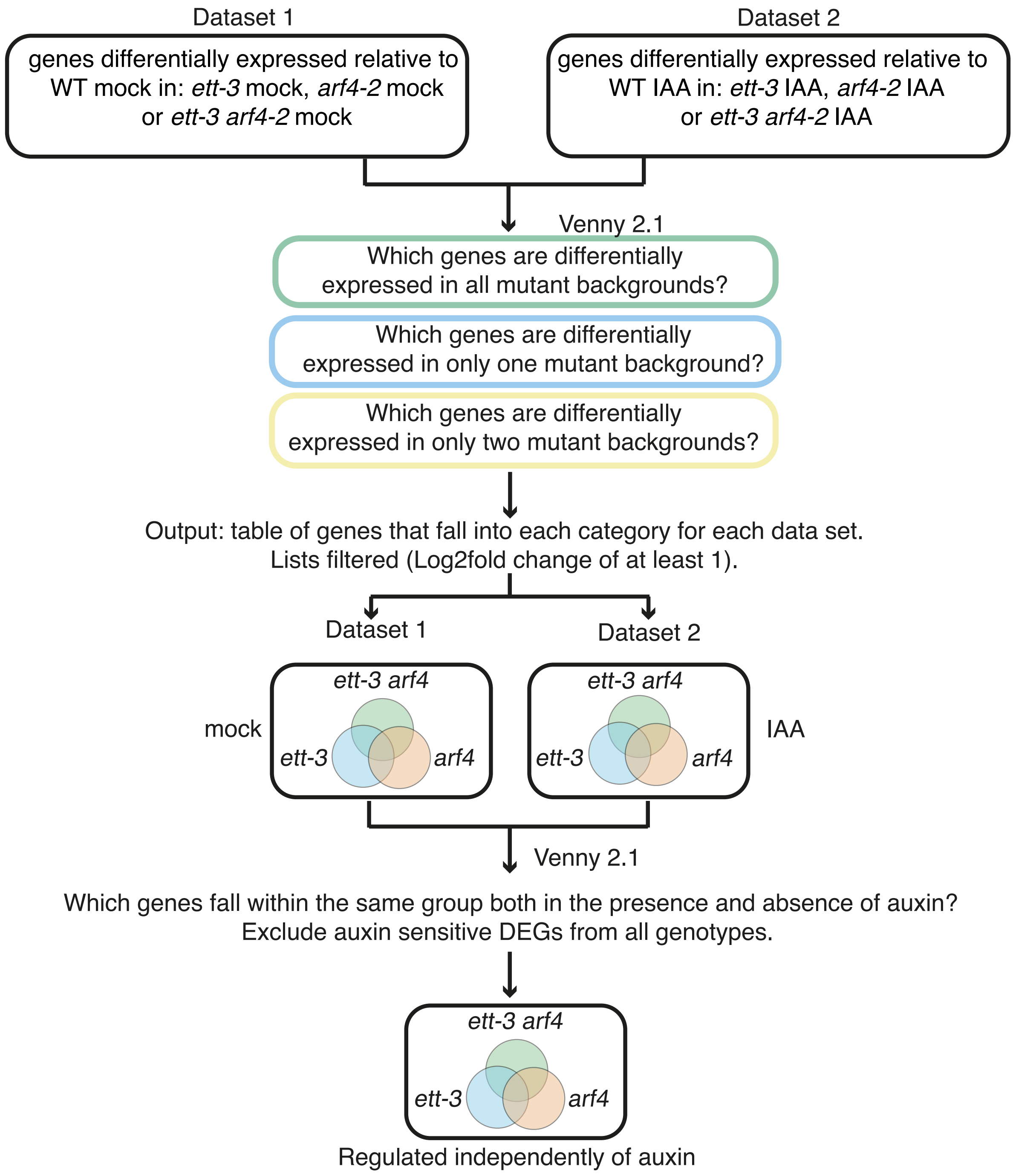

Fig. S3. Schema of auxin independent RNA sequencing analysis. DEG, differentially expressed gene. Two datasets (containing three lists of genes each) were used for this analysis. The first dataset contained lists of genes which were differentially expressed relative to wildtype in *ett-3*, arf4-2 and *ett-3 arf4-2* after mock treatment, whilst the second comprised the same after IAA treatment. The lists were compared in Venny 2.1 to generate two three-way Venn diagrams. Comparisons between the two datasets identified genes which are mis-regulated in the same manner after both mock and auxin treatment. Auxin sensitive genes were removed, leaving genes which are regulated by ETT and ARF4 independently of auxin.

Development • Supplementary information

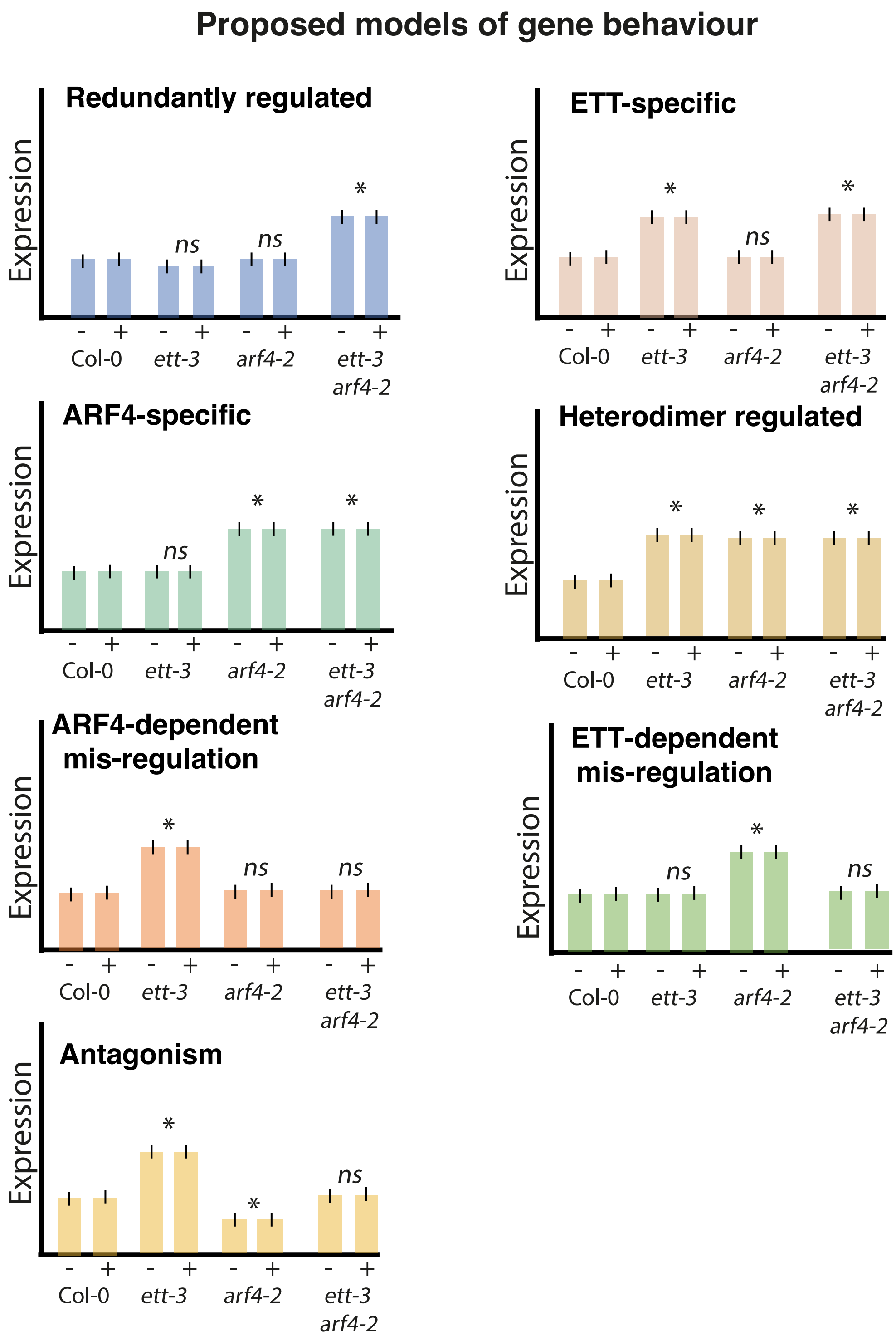

Fig. S4. Proposed models of gene behaviour in each group identified in the auxin sensitive analysis. The * denotes the pattern of significance expected in each group, whilst the genes in the example are up-regulated, they could equally show down-regulation.

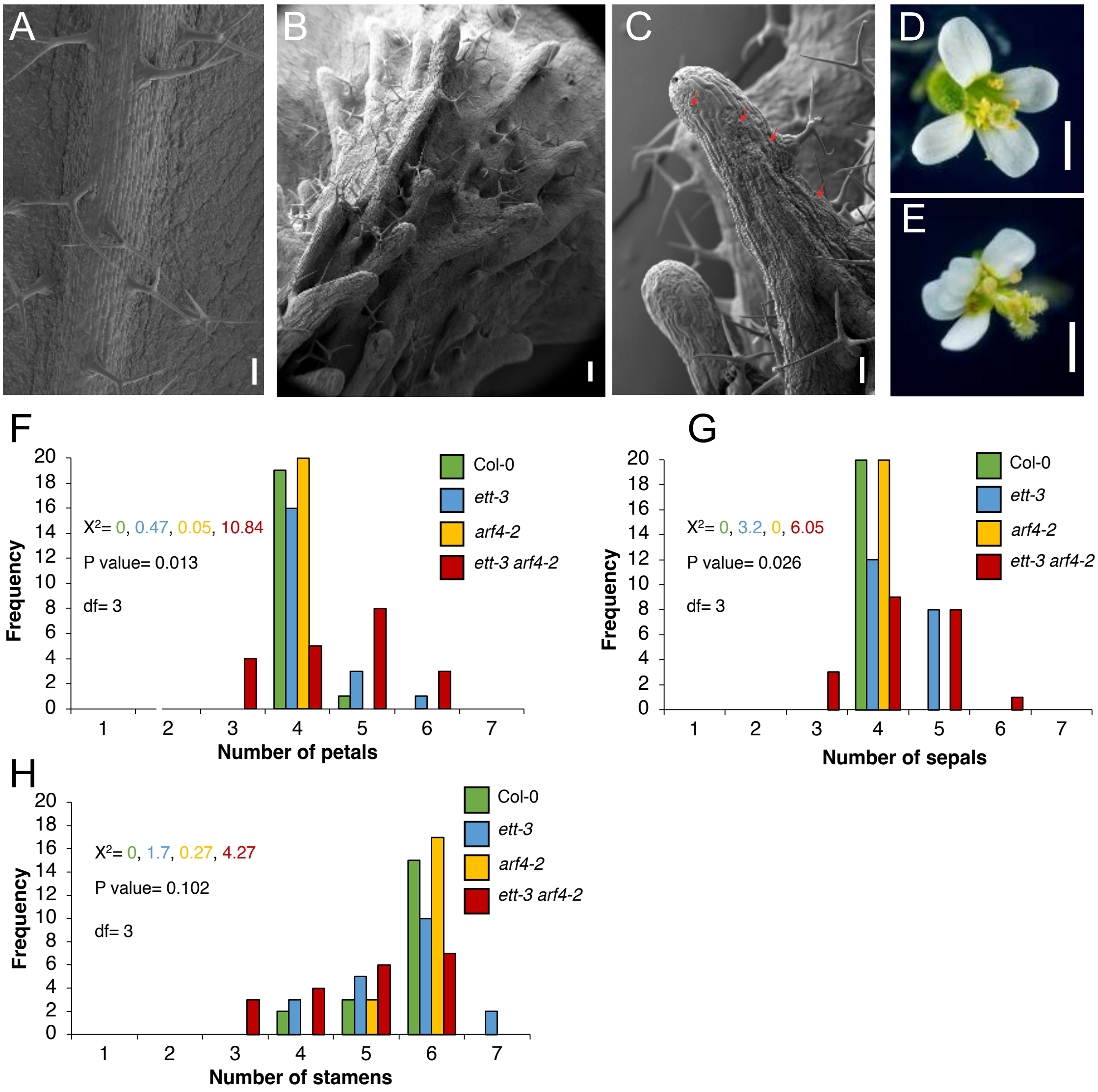

Fig. S5. Phenotypes in mutant lines correlate with the genes mis-regulated in the auxin independent analysis. Scanning electron micrographs of the abaxial surface of: A) Col-0 leaf and midvein, B) and C) *ett-3 arf4-2* leaf and blade like projections formed from midvein-like structures. The red arrows in C show stomata developing on the mid-vein like projections of *ett-3 arf4-2* mutants. Photographs of flowers from Col-0 D), and *ett-3 arf4-2* E). Graphs showing variable floral organ number phenotypes observed for petals F), sepals G), and stamens H) in *ett-3* and *ett-3 arf4-2* mutants. In floral organ count studies, n=20 for all lines. Scale bars A) and B) 200 μm, C) 100 μm, D and E) 1cm.

Development • Supplementary information

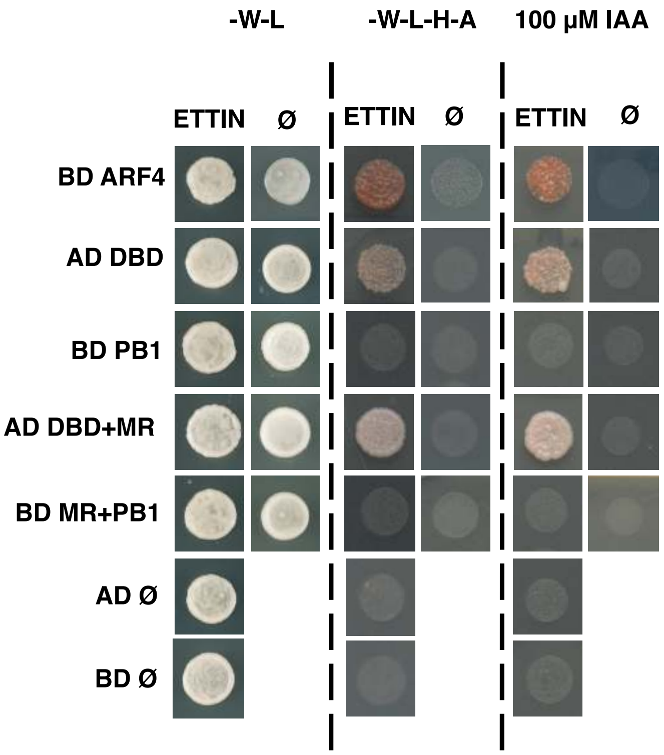

Fig. S6. Yeast 2 hybrid assay assessing the interactions between ETT and ARF4. The assay tested interactions of full length ETT with full length ARF4, the ARF4 DNA binding Domain (DBD, amino acid 0-308), ARF4 PB1 domain (PB1, amino acid 663- 788), the ARF4 DBD and middle region (MR, amino acid 1-663), and the ARF4 MR and PB1 domain (amino acid 308-788), and empty vector controls (ø). -W -L media lacking tryptophan and leucine, -W-L-H-A selection media lacking tryptophan, leucine, histidine, and alanine. Auxin treatment was conducted on the -W-L-H-A media.

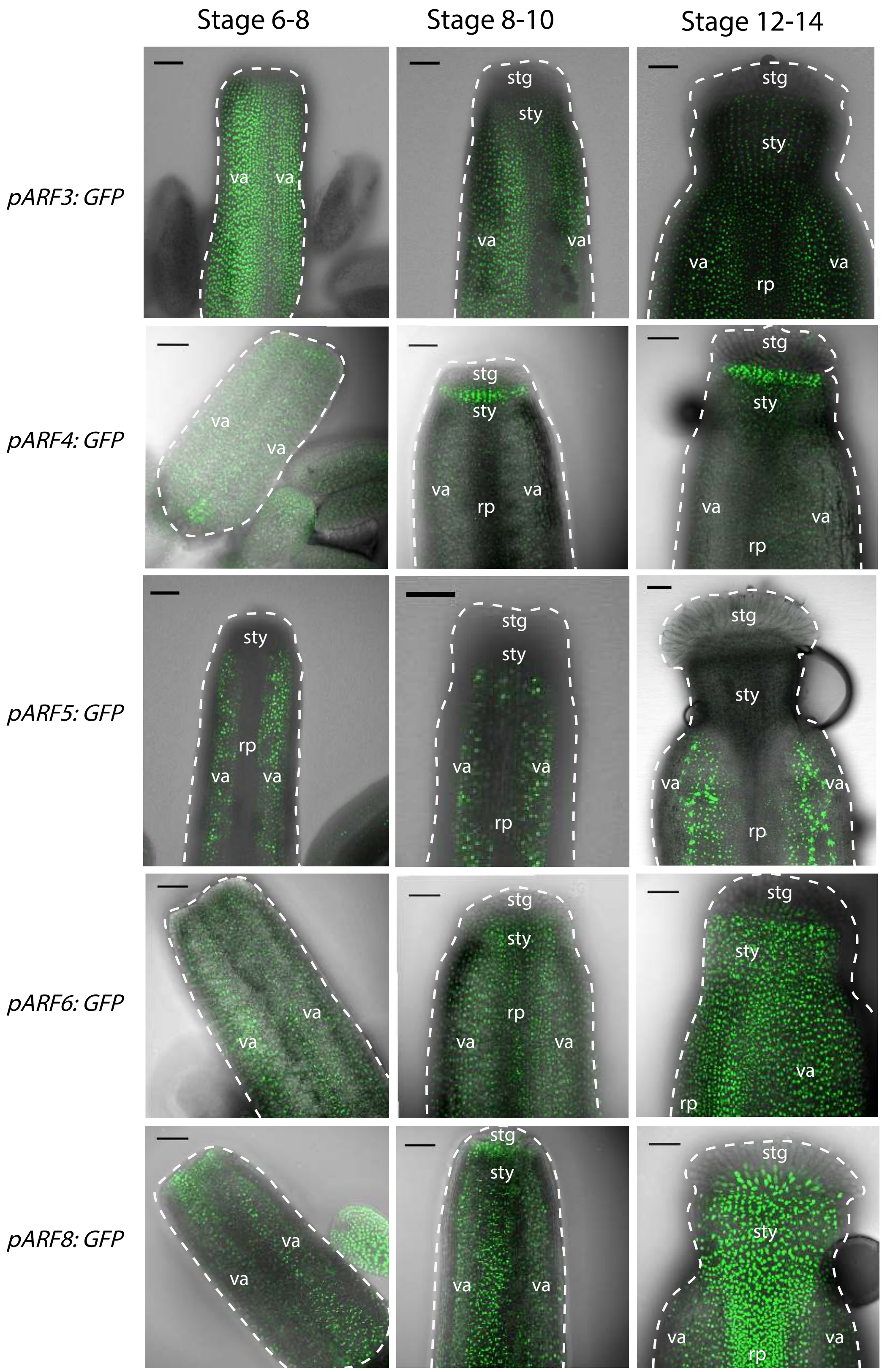

Fig. S7. Expression of *pARF3:GFP*, *pARF4:GFP*, *pARF5:GFP*, *pARF6:GFP and pARF8:GFP* from stage 6-14 of gynoecium development in *Arabidopsis*. Outline of gynoecium in the brightfield image is shown by the dashed line. Va, valve, stg, stigma, rp, replum, sty, style. Scale bars 50 μm.

Development • Supplementary information

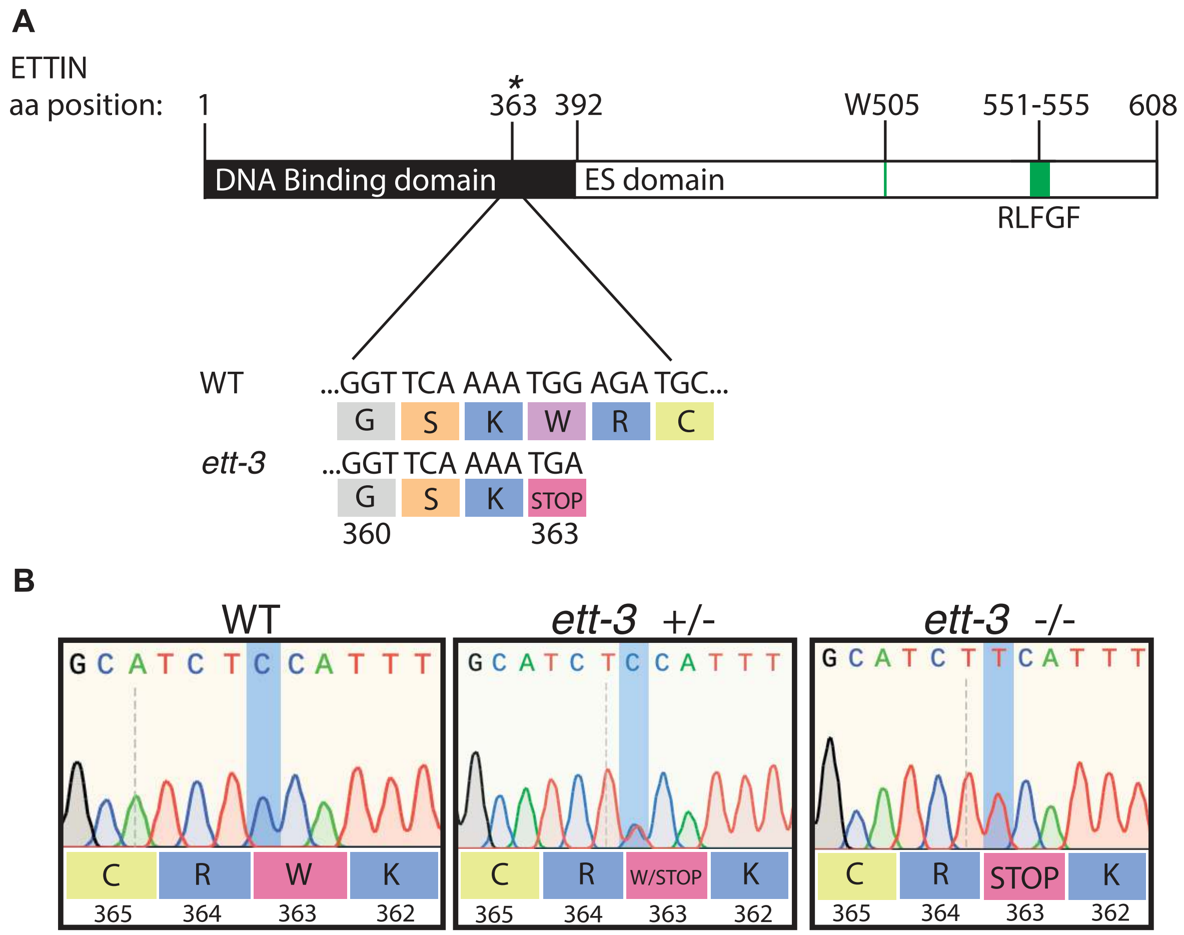

Sequencing read with ETT-R reverse primer

Fig. S8. Summary of the *ett-3* mutation. A) The position in the ETT protein where an early stop codon is introduced in the *ett-3* background. B) The sequencing reads obtained for genotyping the *ett-3* mutation using the ETT-R primer. The mutation is recessive in nature and heterozygotes exhibit no phenotype.

Development • Supplementary information

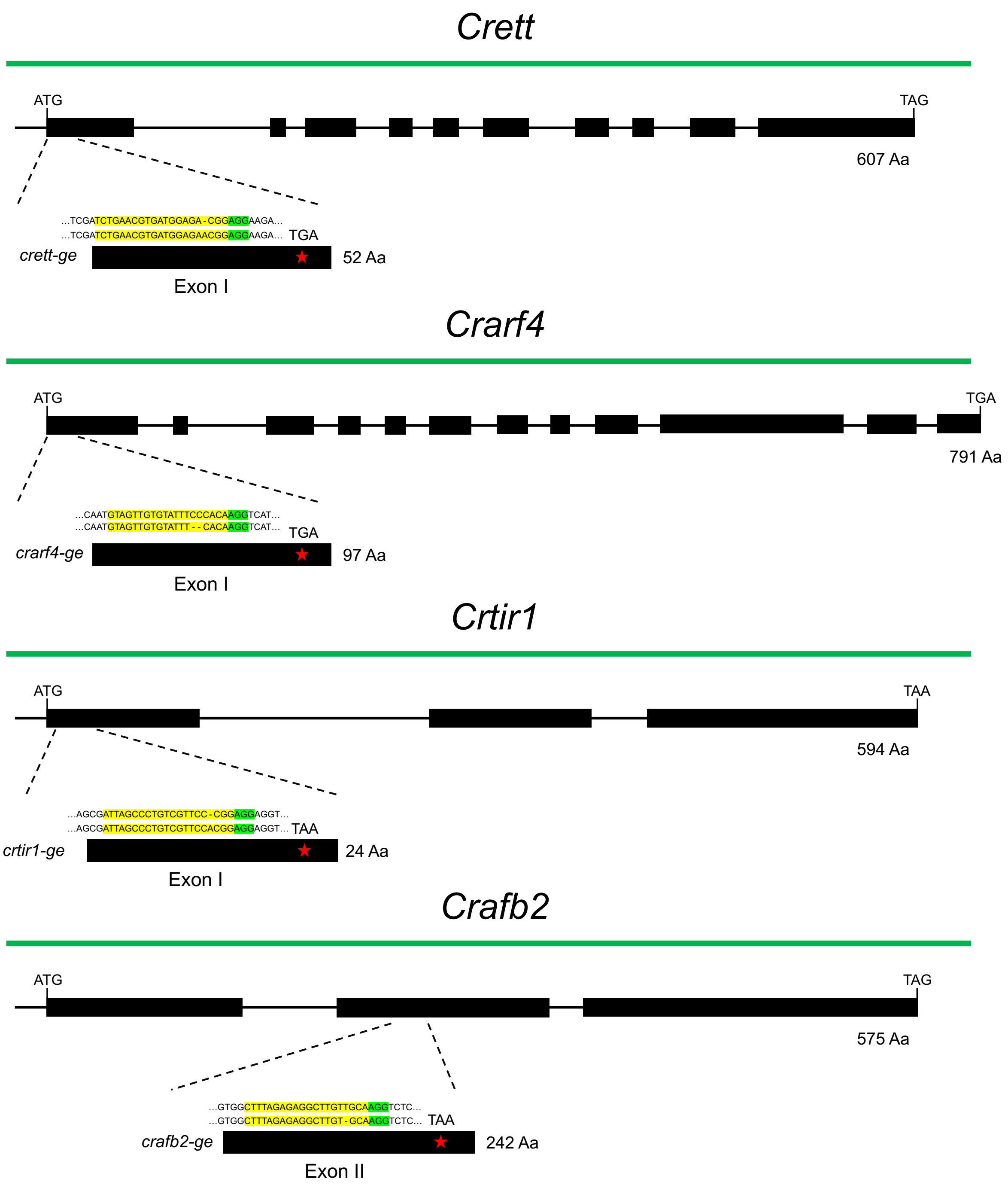

Fig. S9. Information on the mutations induced by CRISPR/Cas9 in *Capsella rubella*. In *Crett*, *Crarf4*, and *Crtir1*, early stop codons were induced by deletions in exon one of the genomic sequence. In *Crafb2*, an early stop codon was produced in exon two. The guide RNAs and PAM sequences were highlighted with yellow and green, respectively.

Development • Supplementary information

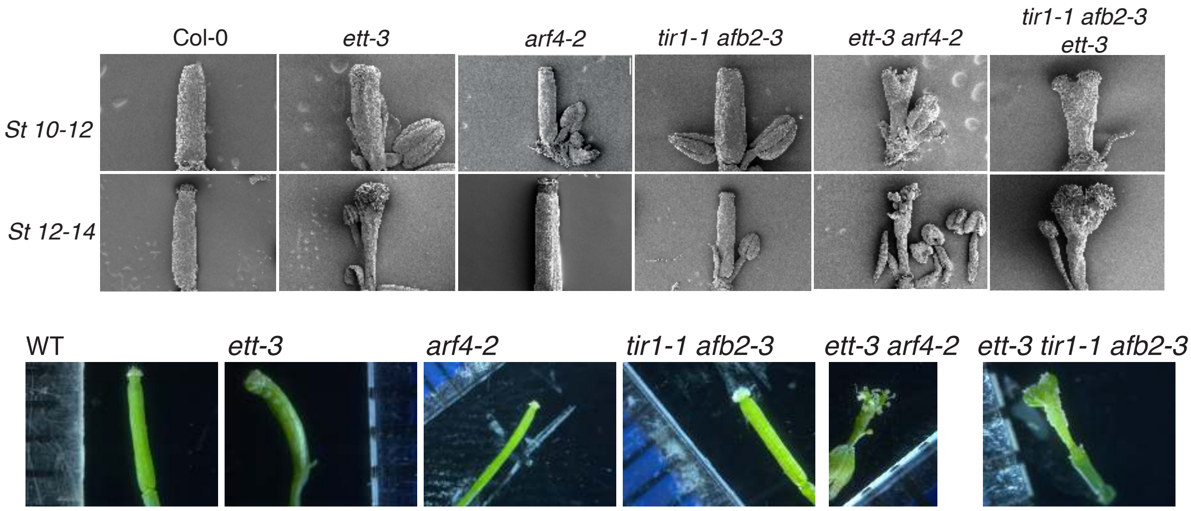

Fig. S10. SEM images (from Fig. 7 A-L) prior to cropping and background removal, scale bars 200 microns. Photographs of stage 16-18 *Arabidopsis* gynoecia before cropping and background clean up (from Fig. 7 M-R).

Development • Supplementary information

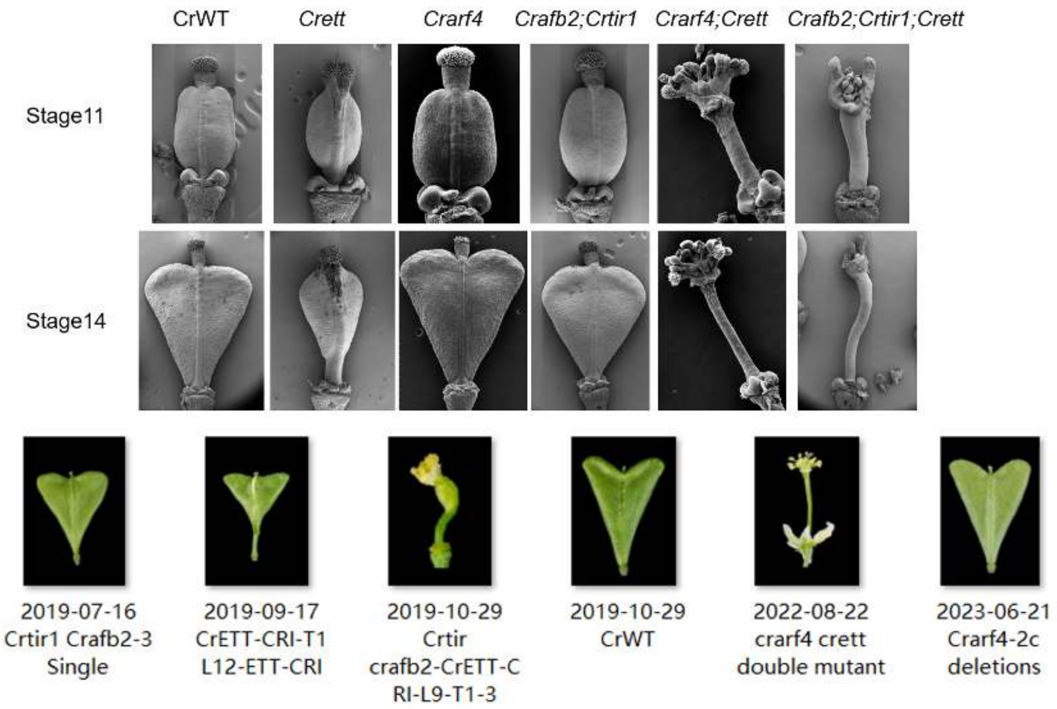

Fig. S11. SEM images stage 11-14 *Capsella* gynoecia (from Fig. 9) prior to cropping and background removal. Original photographs of stage 17 *Capsella* gynoecia from the same figure.

Development • Supplementary information

Table S1. Genes which have previously been reported as auxin sensitive by Paponov *et al.*, 2008 which were also found to be differentially expressed in response to auxin in Col-0 in the RNA-sequencing analysis.

Gene	ATG Number	Log2Fold Value +IAA	p Value	Significance	Process
GH3.3	*AT2G23170*	2.43	1.84E-61	TRUE	Auxin Response
IAA16	*AT3G04730*	0.41	1.15E-06	TRUE	Auxin Signalling
IAA2	*AT3G23030*	1.08	1.49E-11	TRUE	Auxin Signalling
IAA4	*AT5G43700*	0.94	3.46E-04	TRUE	Auxin Signalling
SAUR21	*AT5G18030*	1.42	8.02E-07	TRUE	Auxin Response
SAUR50	*AT4G34760*	0.55	1.51E-05	TRUE	Auxin Response
SAUR64	*AT1G29450*	1.35	1.38E-07	TRUE	Auxin Response
SAUR66	*AT1G29500*	0.74	3.99E-06	TRUE	Auxin Response
SAUR67	*AT1G29510*	0.91	2.75E-09	TRUE	Auxin Response
PIN3	*AT1G70940*	0.43	6.97E-05	TRUE	Auxin Transport
PIN4	*AT2G01420*	0.68	2.36E-07	TRUE	Auxin Transport
ARF10	*AT2G28350*	0.50	1.23E-05	TRUE	Auxin Signalling
CYP79B2	*AT4G39950*	0.60	1.45E-03	TRUE	Auxin Biosynthesis

ARR16	*AT2G40670*	-0.48	9.95E-04	TRUE	Cytokinin Signalling
CKX6	*AT3G63440*	0.73	1.53E-05	TRUE	Cytokinin Degradation
AT5G43450	*AT5G43450*	1.54	6.68E-06	TRUE	Ethylene Biosynthesis
ERF104	*AT5G61600*	-0.60	0.00134427	TRUE	Ethylene Signalling
ERF113	*AT5G13330*	-0.52	0.00023729	TRUE	Ethylene Signalling
ERF114	*AT5G61890*	-1.54	6.58E-07	TRUE	Ethylene Signalling
GA20OX2	*AT5G51810*	0.70	6.46E-05	TRUE	GA Biosynthesis

Development • Supplementary information

Table S2. Statistical assessment of overlap between the Simonini *et al*. 2017 and McLaughlin 2023 datasets (DEG in *ett-3* relative to wildtype after mock treatment). Statistical analysis was conducted using Fisher's Exact Test ($p<0.05$).

**Analysis parameter**	**value**
Overlapping DEGs	1477
p value (Fisher's Exact Test)	$2.8x10^{-146}$
Jaccard Index	0.168
Significance	TRUE
odds ratio	2.746

Table S3. Statistical assessment of overlap between the Simonini *et al*. 2017 and McLaughlin 2023 datasets (DEG in *ett-3* relative to wildtype after IAA treatment). Statistical analysis was conducted using Fisher's Exact Test ($p<0.05$).

**Analysis parameter**	**value**
Overlapping DEGs	1454
p value (Fisher's Exact Test)	$2.4\ x10^{-134}$
Jaccard Index	0.164
Significance	TRUE
odds ratio	2.638

Development • Supplementary information

Table S4. Information on primers, band sizes and optimal T^a for each reaction in *Arabidopsis* line genotyping.

Locus	Type	primers	Genotyping by	Size of product (bp)	Optimal T^a (°C)
tir1-1	EMS	HM86+87	BsaI digestion	WT: 517<br>Mutant: 437 and 80	58
ett-3	EMS	HM39+40	sequencing	246	46
arf4-2	SALK	WT:HM27+28,<br>Mutant:HM28+LBb1.3	SALK PCRs	WT: 1196<br>Mutant: 577-877	58<br>58
afb2-3	SALK	WT: HM31+32<br>Mutant:<br>HM32+LBb1.3	SALK PCRs	WT: 1131<br>Mutant:598-898	56<br>58

Table S5. Primer sequences used for genotyping *Arabidopsis* lines and generation of Y2H constructs in this study.

Primer	Sequence	Used for
HM86	GCGAATAGCCTTGTCGTTTCC	genotyping
HM87	TCCTAGAGACACAAGGGAGTCTT	genotyping
HM39	CAGATCCCCTGGGATTATAAGTGG	genotyping
HM40	AGACACGCTGTTGATGCCCATTTG	genotyping
HM27	GTGCGTCCTCTCATCTTTTTG	genotyping
HM28	CGCGACAAATCAATAGCTCTC	genotyping
HM31	TCAACGGTCAAGATCCATCTC	genotyping
HM32	CTGCAATTAGCGGCAATAGAG	genotyping
LBb1.3 (T-DNA)	ATTTTGCCGATTTCGGAAC	genotyping
attB1_ARF4_PB1_F1	GGGGACAAGTTTGTACAAAAAAGCAGGCTCCAAAAGAATCTGTACAAAGGTT	Amplifying PB1 domain ARF4 Y2H
attB2_ARF4_DBD_1R	GGGGACCACTTTGTACAAGAAAGCTGGGTCTGTAGATACAGCATTAGCCAC	Amplifying DBD ARF4 Y2H

Development • Supplementary information

attB2_ARF4_MR_R1	GGGGACCACTTTGTACAAGAAAGCTGGGTCGCTCGAGCTTTGCGGCTTAG	Amplifying ARF4 DBD and MR Y2H
attB1_ARF4_MR_PB1_1F	GGGGACAAGTTTGTACAAAAAAGCAGGCTCC GCCAAGAAATGGACTTCCTGACTCA	Amplifying ARF4 MR and PB1 domain Y2H
attB2_ARF4_MR_PB1_1R	GGGGACCACTTTGTACAAGAAAGCTGGGTC TCAAACCCTAGTGATTGTAGGAGAAG	Amplifying ARF4 MR and PB1 domain Y2H
attB1_ETT_1F	GGGGACAAGTTTGTACAAAAAAGCAGGCTCCATGGGTGGTTTAATCGATCTG	Amplifying Full Length ETT Y2H
attB2_ETT_1R	GGGGACCACTTTGTACAAGAAAGCTGGGTCCTAGAGAGCAATGTCTAGCAACA	Amplifying Full Length ETT Y2H
attB1_ARF4_1F	GGGGACAAGTTTGTACAAAAAAGCAGGCTCCATGGAATTTGACTTGAATACT	Amplifying Full Length ARF4 Y2H
attB2_ARF4_1R	GGGGACCACTTTGTACAAGAAAGCTGGGTC TCAAACCCTAGTGATTGTAG	Amplifying Full Length ARF4 Y2H

Development • Supplementary information

Table S6. List of primers used for the construction of GUS reporter lines, genome editing and genotyping in *Capsella*.

Primer	Sequence (5' to 3')	Used in experiment
pCrAFB2-F	*CCTCTAGAGTCGACCTGCAGTCAA CCTACTGACTACGTATC	GUS line construction
pCrAFB2-R	*CTCAGATCTACCATGGCTCTCCAG TAACTTGAACAAC	GUS line construction
pCrAFB3-F	*CCTCTAGAGTCGACCTGCAGGAG TTTTATTATGAAGCCTTT	GUS line construction
pCrAFB3-R	*CTCAGATCTACCATGGCTTCCGAA ACCCAAACAATAGG	GUS line construction
pCrTIR1-F	*CCTCTAGAGTCGACCTGCAGTGAC CTCTCTAATCGCATCC	GUS line construction
pCrTIR1-R	*CTCAGATCTACCATGGTGCGCCCA AGAATAACTTCG	GUS line construction
pCrETT-F	*CCTCTAGAGTCGACCTGCAGAAA ACGCACCAAGTAATTATGA	GUS line construction
pCrETT-R	*CTCAGATCTACCATGGTATAGAG AGAGAGACAGAGATA	GUS line construction
pAtAFB2-F	CCTCTAGAGTCGACCTGCAGATTG TCTTGAATAATTGTTTCATCCATA	GUS line construction
pAtAFB2-R	*CTCAGATCTACCATGGCTCTAACT TCACCAGCAAGATTCC	GUS line construction

pAtAFB3-F	*CCTCTAGAGTCGACCTGCAGCAC ATGGCAGCCATTGTAGC	GUS line construction
pAtAFB3-R	*CTCAGATCTACCATGGCTTCCTCT ATTGATTGTCGAACAGAG	GUS line construction
pAtTIR1-F	*CCTCTAGAGTCGACCTGCAGTCAT TCAGAGTTGGATCTAATATCGCT	GUS line construction
pAtTIR1-R	*CTCAGATCTACCATGGTGCGGCCA AATAACCTCG	GUS line construction
CrAFB2-Exon2-gRNA1	CTTTAGAGAGGCTTGTTGCA	Gene editing
CrAFB3-Exon1-gRNA1	GAACTGCTACGCCATCAACC	Gene editing
CrTIR1-Exon1-gRNA1	ATTAGCCCTGTCGTTCCCGG	Gene editing
CrARF4-Exon1-gRNA1	GTAGTTGTGTATTTCCCACA	Gene editing
CrETT-Exon1-gRNA1	TCTGAACGTGATGGAGACGG	Gene editing
CrAFB2-Geno-F	GCATCTTCGGGATCTGGACTTG	Gene CRISPR genotyping
CrAFB2-Geno-R	TTTCTTTATGGCAGCCTTGAGT	Gene CRISPR genotyping
CrAFB3-Geno-F	TCATCGCTTCTCACAGAGACA	Gene CRISPR genotyping
CrAFB3-Geno-R	AATGGAAGCTAAGCCGTCAG	Gene CRISPR genotyping
CrTIR1-Geno-F	TTCGGACTTCTTTCCCCAACTCTG	Gene CRISPR genotyping
CrTIR1-Geno-R	TCAGCGAAGTGAGGTTTCCCCT	Gene CRISPR genotyping
CrETT-Geno-F	TGCTAGTCTCTCTTCTTTTGGCT	Gene CRISPR genotyping
CrETT-Geno-R	AACAAAACAAACCCCCATAC	Gene CRISPR genotyping
CrARF4-Geno-F	CCTCTTCCACAGGCTCTGCCTC	Gene CRISPR genotyping
CrARF4-Geno-R	AGGCGTTCGTTTAACGGTTGAG	Gene CRISPR genotyping

*The sequences shaded in yellow indicate the homologs to the plasmid and used for In-Fusion cloning.

Development • Supplementary information

Table S7. FPKMs of all genes expressed in this study

Available for download at
https://journals.biologists.com/dev/article-lookup/doi/10.1242/dev.204263#supplementary-data

Table S8. GO term analyses conducted in this study

Available for download at
https://journals.biologists.com/dev/article-lookup/doi/10.1242/dev.204263#supplementary-data

Table S9. Results of the auxin independent analysis conducted in this study

Available for download at
https://journals.biologists.com/dev/article-lookup/doi/10.1242/dev.204263#supplementary-data

Table S10. Results of the auxin sensitive analysis conducted in this study

Available for download at
https://journals.biologists.com/dev/article-lookup/doi/10.1242/dev.204263#supplementary-data

Development • Supplementary information
