# Supplementary material for: Conserved roles of ETT and ARF4 in gynoecium development in Brassicaceae with distinct fruit shapes
Source: Development. 2025 Feb 12;152(3):DEV204263. doi: 10.1242/dev.204263 (PMC11883277; doi:10.1242/dev.204263)
Supplement: Supplementary file 1 [file develop-152-204263_review_history.pdf]

Conserved roles of ETT and ARF4 in gynoecium development in Brassicaceae with distinct fruit shapes

Heather Marie McLaughlin, Tian-Feng Lü, Bhavani Natarajan, Lars Østergaard and Yang Dong
DOI: 10.1242/dev.204263

Editor: Liam Dolan

Review timeline

Original submission:	5 January 2024
Editorial decision:	1 March 2024
Resubmission:	22 July 2024
Editorial decision:	18 October 2024
First revision received:	19 December 2024
Accepted:	6 January 2025

Original submission

First decision letter

MS ID#: DEVELOP/2024/202683

MS TITLE: Conserved auxin signalling synergism through the canonical and ETT-mediated pathways promotes gynoecium development in members of the Brassicaceae

AUTHORS: Heather Marie McLaughlin, Tian-Feng Lv, Bhavani Natarajan, Lars Østergaard , and Yang Dong

I have now received all the referees reports on the above manuscript, and have reached a decision. The referees' comments are appended below, or you can access them online: please go to BenchPress and click on the 'Manuscripts with Decisions' queue in the Author Area.

As you will see from their reports, the referees recognise the potential of your work, but they also raise significant concerns about it. Given the nature of these concerns, I am afraid I have little choice other than to reject the paper at this stage.

However, having evaluated the paper, I do recognise the potential importance of this work. I would therefore be prepared to consider as a new submission an extension of this study that contains new experiments, data and discussions and that address fully the major concerns of the referees. The work required goes beyond a standard revision of the paper. Please bear in mind that the referees (who may be different from the present reviewers) will assess the novelty of your work in the context of all previous publications, including those published between now and the time of resubmission.

© 2025. Published by The Company of Biologists under the terms of the Creative Commons Attribution License (https://creativecommons.org/licenses/by/4.0/).

Both referees have raised issues that - if not addressed - would lead me to reject the paper. One refers to the nature of the ettin mutant, and the need for an analysis of a complete loss of function mutant. The other is that the manuscript seems to be made of two incomplete "stories" neither of which are complete. If you can address these issues, I would consider sending this for a second round of reviews. However, if you cannot deal with these comments, I would understand if you submitted your manuscript elsewhere.

Reviewer 1

Advance summary and potential significance to field

The authors explored the potential redundancy between the previously reported "non-canonical" role that ARF3/ETT plays in gynoecium development and TIR1/AFB-dependent "canonical" signaling. While the data appears to be of high quality, I feel that these are two stories stitched together. A story related to comparative expression patterns of ETT and TIR1/AFB genes and mutant phenotypes across Capsella and Arabidopsis and a second story about the set of genes regulated by ETT and/or ARF4 in Arabidopsis. Each of these are interesting stories with valuable findings, but both appear incomplete and there is little connection or added value in presenting these together. This could for example have been achieved by including RNAseq in the Capsella mutants or exploration of the conserved function of selected differentially expressed genes.
Each part of the manuscript separately appears somewhat cursory. The co-expression analysis lacks the tissue-scale resolution that is required to conclude about shared function. The phenotypic analysis (SEM) is too course-grained to know whether the same developmental processes are targeted in the two species. The RNAseq analysis is interesting but provides a list of genes that is not explored for biological relevance.

I would encourage the authors to either develop each story separately or develop a better connection between the parts so that one conceptual message is delivered.

Comments for the author

See above

Reviewer 2

Advance summary and potential significance to field

The ms. by McLaughlin et al brings two main advances to the field:
1. The generation of Capsella rubella mutants in ETT, ARF4, TIR1 and AFB2 loci. These mutants are useful to assess the conservation of these functions in another Brassicaceae species that has already been shown to have a characteristic fruit morphology related to auxin signaling modification respect to Arabidopsis. A number of useful reporter lines both in Arabidopsis and Capsella are also a product of this work.
2. An assessment of the potential contribution of canonical and non-canonical (mediated by ETT) auxin signaling in gynoecium development through genetic and transcriptomic analyses. These results also lead to the identification of downstream targets of ETT and ARF4. Both major contributions are of high interest for researchers working in flower development and also in more general understanding of auxin importance and mechanisms of action in morphogenesis

Comments for the author

© 2025. Published by The Company of Biologists under the terms of the Creative Commons Attribution License (https://creativecommons.org/licenses/by/4.0/).

In my opinion the paper is very interesting and provides significant and interesting results.
The workflow and rationale of the transcriptomic experiments are clever and well developed and could inspire similar approaches in the field, in addition to provide the information sought in this work, which is highly valuable.
However, I think the paper has one major flaw, the use of ett-3 allele for the analysis. I really do not understand this choice. ett-3 is not a null allele, it has a stronger-than-null effect caused by a missense mutation in the second half of the protein. I have not been able to find whether this affects or not the direct interaction of ETT with auxin (or the possible conformational change induced by this interaction) that is at the root of the non-canonical auxin signaling. In any case, it would have been very easy to carry out the experiments using an ett null allele and the results would be much more solid and less prone to misinterpretations. I understand that repeating all the analysis at this point with an ett null is quite a lot, but in my opinion, the study would be much better. An alternative would be to repeat the transcriptomic analyses with the Capsella rubella mutants already at hand (just the transcriptomics, no need for validation of targets if a significant overlap is found) and see if the conclusions hold and it is really possible to segregate canonical and non-canonical signaling components in gynoecium development.

Other than this, which I think is a major issue, I do not find other significant points for improvement.

First revision

Author response to reviewers' comments

Review Round 1:

Reviewer 1
The authors explored the potential redundancy between the previously reported "non- canonical" role that ARF3/ETT plays in gynoecium development and TIR1/AFB-dependent "canonical" signaling. While the data appears to be of high quality, I feel that these are two stories stitched together. A story related to comparative expression patterns of ETT and TIR1/AFB genes and mutant phenotypes across Capsella and Arabidopsis and a second story about the set of genes regulated by ETT and/or ARF4 in Arabidopsis. Each of these are interesting stories with valuable findings, but both appear incomplete and there is little connection or added value in presenting these together. This could for example have been achieved by including RNAseq in the Capsella mutants or exploration of the conserved function of selected diLerentially expressed genes. Each part of the manuscript separately appears somewhat cursory. The co-expression analysis lacks the tissue-scale resolution that is required to conclude about shared function. The phenotypic analysis (SEM) is too course- grained to know whether the same developmental processes are targeted in the two species. The RNAseq analysis is interesting but provides a list of genes that is not explored for biological relevance. I would encourage the authors to either develop each story separately or develop a better connection between the parts so that one conceptual message is delivered.

Response: We agree with the reviewer that the connection between the two parts of the story could be improved. In the light of this suggestion, we have redrafted the manuscript to clarify the connection between the parts of the story, and so that one cohesive narrative comes through. We feel that the manuscript has greatly benefitted from these changes.

Reviewer 2
In my opinion the paper is very interesting and provides significant and interesting results. The workflow

© 2025. Published by The Company of Biologists under the terms of the Creative Commons Attribution License (https://creativecommons.org/licenses/by/4.0/).

and rationale of the transcriptomic experiments are clever and well developed and could inspire similar approaches in the field, in addition to provide the information sought in this work, which is highly valuable. However, I think the paper has one major flaw, the use of ett-3 allele for the analysis. I really do not understand this choice. ett-3 is not a null allele, it has a stronger-than-null effect caused by a missense mutation in the second half of the protein. I have not been able to find whether this affects or not the direct interaction of ETT with auxin (or the possible conformational change induced by this interaction) that is at the root of the non-canonical auxin signaling. In any case, it would have been very easy to carry out the experiments using an ett null allele and the results would be much more solid and less prone to misinterpretations. I understand that repeating all the analysis at this point with an ett null is quite a lot, but in my opinion, the study would be much better. An alternative would be to repeat the transcriptomic analyses with the Capsella rubella mutants already at hand (just the transcriptomics, no need for validation of targets if a significant overlap is found) and see if the conclusions hold and it is really possible to segregate canonical and non-canonical signaling components in gynoecium development.

Response: Thank you for raising the concern on the *ett-3* mutant. The *ett-3* allele produces a recessive medium strength phenotype which is less strong than e.g. *ett-1* (with a T-DNA insertion in exon 2). This can be observed in Fig. 1 and 2 of Sessions et al. 1997 (Development, 124, 4481-4491). The *ett-3* allele is not a mis-sense allele, but a non-sense allele causing a premature stop codon where tryptophan 363 would otherwise be located.
This is upstream of the ETT-Specific (ES) domain (amino acids 392-609) responsible for auxin binding and containing the EAR-like (RLFGF) motif required for ETT to interact with TPL (Simonini et al., 2016; 2018; Kuhn et al., 2020). The auxin-binding region and TPL- interacting regions of the protein (which underly its auxin sensitivity) are therefore not produced in the *ett-3* mutant, making it a suitable allele to observe what happens when ETT- mediated signalling is disabled. Indeed, this is the allele that has been used to unravel the non-canonical auxin signalling pathway mediated by ETT (Simonini et al., 2016/2017 and Kuhn et al. 2020). We therefore feel that the use of the *Arabidopsis ett-3* allele is justified in this study.
In response to the reviewer's statement, and to make this absolutely clear, we have added text in the manuscript which discusses the *ett-3* allele in relation to the auxin binding and TPL-associating regions of the protein. We have also added an additional Supplementary Figure highlighting the position of the premature stop codon in relation to the other regions of importance in the ETT protein, the nature of the mutation and its genotyping.

Resubmission

MS ID#: dev.204263

MS TITLE: Conserved roles of ETT and ARF4 in gynoecium development in Brassicaceae with distinct fruit shapes

AUTHORS: Yang Dong, Heather Marie McLaughlin, Tian-Feng Lü, Bhavani Natarajan and Lars Østergaard

Firstly, please accept my apologies for the delayed response on this manuscript. We were trying to get feedback from the initial referees, but unfortunately have only been received one report, despite numerous reminders. To avoid further delays, however, we are taking a decision based on the feedback we have (which you will find appended below, or you an access them online via).
I am sorry to say that the referee still raises significant concerns with the paper, that were not fully addressed in your revisions. Having discussed this in detail with Liam Dolan, the handling editor for

© 2025. Published by The Company of Biologists under the terms of the Creative Commons Attribution License (https://creativecommons.org/licenses/by/4.0/).

your paper, we do feel that the requests by the referees are important for the conclusions of your paper and are therefore returning it to you for further revision. While we do normally aim to limit revisions to a single major round, we are in this case prepared to consider a further revision as we do see the potential in this work - provided you are able to address the key concerns of the referee. If, however, you prefer to take the paper elsewhere at this point, please let us know so we can remove your paper from the active area of our submission system.
If it would be helpful, you are welcome to contact us to discuss your revision in greater detail. Please send us a point-by-point response indicating your plans for addressing the referees' comments, and we will look over this and provide further guidance.

Please attend to all of the reviewers' comments and ensure that you clearly highlight all changes made in the revised manuscript. Please avoid using 'Tracked changes' in Word files as these are lost in PDF conversion. I should be grateful if you would also provide a point-by-point response detailing how you have dealt with the points raised by the reviewers in the 'Response to Reviewers' box. If you do not agree with any of their criticisms or suggestions please explain clearly why this is so.

Comments from the Reviewers:
Reviewer 1: The manuscript by McLaughlin et al. is an interesting study focused on understanding the redundant and specific functions of ETT and ARF4, both dependent and independent of auxin, in the context of gynoecium development. Most of the results rely on a clever transcriptomic analysis strategy designed to differentiate the common and specific roles of ARF4 and ETT, particularly those independent of auxin. I previously reviewed an earlier version of this manuscript that was submitted a few months ago. Although this is an independent submission, I find myself missing a response to the concerns raised by reviewers in the earlier submission. That said, I believe this version is more focused on the Arabidopsis results and has shown some improvement. However, I still have concerns regarding the ett-3 allele, which I had pointed out in my earlier review. The authors assert that the truncated ETT-3 protein lacks the domain necessary for interaction with TPL and auxin. While this is interesting, it seems to contradict the framework of the transcriptomic analyses, as it is unclear to me whether the truncated protein retains any auxin-independent functions, given the stronger-than-null phenotype it exhibits. Could it be possible that the truncated ETT-3 interacts with ARF4 and disrupts the system? Could the truncated protein still bind to DNA (and potentially block ARF4)? The results from Capsella could support these possibilities. I do not understand why keep using the ett-3 allele, really, for all these works.

Additionally, I have concerns about the relatively limited overlap between the RNA-seq data presented in this study and those from Simonini (2017). While the authors offer a potential explanation, this discrepancy remains puzzling. Including data on ETT direct targets (from ChIP-seq) within the analysis would be valuable, as these targets are crucial for providing mechanistic insights into the specificity of ETT and ARF4 in the presence or absence of auxin, which is central to this study. Furthermore, incorporating transcriptomic analyses of the equivalent mutants in Capsella would significantly strengthen the paper's conclusions.
In summary:
* To gain confidence in the interpretation of the results, transcriptomic analysis including an ett null allele (which I understand would require substantial additional work) or transcriptomics in the Capsella mutants would be necessary.
* Investigating the interaction between the truncated ETT-3 protein and ARF4, as well as its interaction with chromatin, could provide further clarity in interpreting the results.
* Overlapping previously identified ETT direct targets with the current transcriptomic analyses would also be a valuable addition.

© 2025. Published by The Company of Biologists under the terms of the Creative Commons Attribution License (https://creativecommons.org/licenses/by/4.0/).

First revision

Author response to reviewers' comments

No response from Reviewer 1.

Review Round 2:

Response to Reviewer 2, point by point:

Thank you for the response and for the opportunity to again provide additional clarification on our work. Our responses are highlighted below in blue.

Reviewer: "I believe this version is more focused on the Arabidopsis results and has shown some improvement." We have significantly redrafted the manuscript in line with previous requests of reviewer 1 so that a clear narrative comes through, as the reviewer stated previously that the connection between parts of the paper seemed unclear In the previous version. This redraft has clarified how the distinct parts of the manuscript relate to one another. We feel that this makes the story much more cohesive. Reviewer 1 requested either more experiments or a significant redraft- we chose to conduct a significant redraft.

Reviewer: "However, I still have concerns regarding the ett-3 allele, which I had pointed out in my earlier review. The authors assert that the truncated ETT-3 protein lacks the domain necessary for interaction with TPL and auxin. While this is interesting, it seems to contradict the framework of the transcriptomic analyses, as it is unclear to me whether the truncated protein retains any auxin-independent functions, given the stronger-than- null phenotype it exhibits. Could it be possible that the truncated ETT-3 interacts with ARF4 and disrupts the system? Could the truncated protein still bind to DNA (and potentially block ARF4)? The results from Capsella could support these possibilities. I do not understand why keep using the ett-3 allele, really, for all these works."

As we stated in the previous round of review, the ett-3 allele is a well-established (Sessions and Zambryski, 1995; Sessions et al., 1997; Simonini et al., 2016; Simonini et al., 2017; Kuhn et al., 2020) allele of intermediate phenotype (Sessions et al., 1997) caused by a non-sense mutation and premature stop codon where tryptophan 363 would otherwise be located.

It is not a null mutant as the Reviewer rightly points out, but we have added a discussion on the nature of the mutation in the paper and have added additional supplemental Figures (Figure S8, A and B) to show the nature of the mutation and how it relates to the structure of the protein. The reviewer mentions a "stronger than null phenotype" which we do not observe- we are not sure why the reviewer thinks this. We have provided a side- by-side comparison of the Arabidopsis ett-3 with a null, ett-22, and see that the phenotypes are similar between the two lines. We see no evidence of a "stronger than null" effect.

© 2025. Published by The Company of Biologists under the terms of the Creative Commons Attribution License (https://creativecommons.org/licenses/by/4.0/).

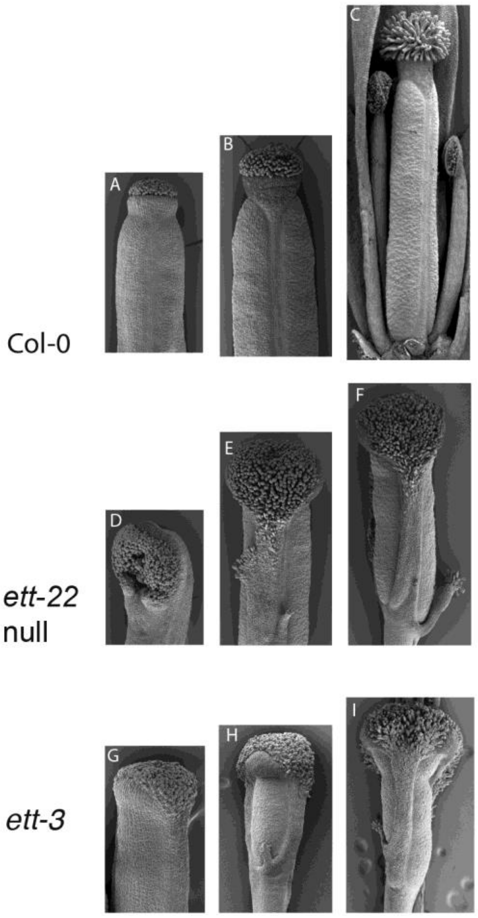

Review Fig. 1: Comparative view of representative phenotypes produced in gynoecia of Col-0, *ett-22* and *ett-3* lines. Images A-B and D-F were provided by Emilie Knight, JIC (Østergaard Lab) Images G-I by Heather McLaughlin (Østergaard Lab) and C is previously published in Girin et al. 2011 *Plant Cell* https://doi.org/10.1105/tpc.111.090944

Reviewer: "Additionally, I have concerns about the relatively limited overlap between the RNA-seq data presented in this study and those from Simonini (2017). While the authors oLer a potential explanation, this discrepancy remains puzzling. Including data on ETT direct targets (from ChIP-seq) within the analysis would be valuable, as these targets are crucial for providing mechanistic insights into the specificity of ETT and ARF4 in the presence or absence of auxin, which is central to this study. Furthermore, incorporating transcriptomic analyses of the equivalent mutants in Capsella would significantly strengthen the paper's conclusions."

We refer the Reviewer to Figure S2 and Table S2 and S3 which show there is significant overlap between the data presented in this manuscript and the study from Simonini et al., 2017. The discrepancies between studies were in very few individual genes and are discussed in the text of the results section.

We agree that future studies looking into the direct vs indirect targets would be useful, but this is beyond the scope of the current study, it would require extensive funding and resources which are not available. We have added a section to the discussion highlighting that this would

© 2025. Published by The Company of Biologists under the terms of the Creative Commons Attribution License (https://creativecommons.org/licenses/by/4.0/).

be a pertinent question in the future. We refer the Reviewer to the discussion which states "Future studies should aim to determine which of the targets identified within this study are direct/indirect ETT/ARF4 targets, and to validate the roles of the targets identified in the regulation of gynoecium development in Arabidopsis and Capsella."

Reviewer
"In summary:

* To gain confidence in the interpretation of the results, transcriptomic analysis including an ett null allele (which I understand would require substantial additional work) or transcriptomics in the Capsella mutants would be necessary."
Given that the phenotype in the ett-3 is very similar to the null, and we see no evidence of truncated ETT activity, we feel that these analyses are not necessary in the current paper. There are strengths and weaknesses to the use of many mutant lines, and we discuss the nature of the allele used in the paper. Given that there is no evidence of a "stronger than null" effect, we feel that the additional experiments are not required in this case.

"* Investigating the interaction between the truncated ETT-3 protein and ARF4, as well as its interaction with chromatin, could provide further clarity in interpreting the results." Again, we see no evidence of these and feel therefore that this is not necessary. Looking into chromatin interactions is beyond the scope of this paper but would make interesting future work.

"* Overlapping previously identified ETT direct targets with the current transcriptomic analyses would also be a valuable addition."
We do see significant overlap between our current study and the Simonini et al 2017 dataset. We have discussed the lack of data around what is direct vs indirect in the discussion but cannot conduct further experiments or extensive analyses, as the funding for the project has concluded.

<u>Second decision letter</u>

MS ID#: dev.204263R1

MS TITLE: Conserved roles of ETT and ARF4 in gynoecium development in Brassicaceae with distinct fruit shapes

AUTHORS: Yang Dong, Heather Marie McLaughlin, Tian-Feng Lü, Bhavani Natarajan and Lars Østergaard

I am happy to tell you that your manuscript has been accepted for publication in Development, pending our standard publication integrity checks. It was accepted on 06 Jan 2025. Where referee reports on this version are available, they are appended below. Please do accept our apologies for the long delays in handling your manuscript, but I'm delighted that we were able to get additional feedback and that we're now in a position to accept it for publication.

Comments from the Reviewers:

© 2025. Published by The Company of Biologists under the terms of the Creative Commons Attribution License (https://creativecommons.org/licenses/by/4.0/).

Reviewer 2: This manuscript has three components: a transcriptomic analysis of ett/arf3, arf4 and ett/arf4 double mutants, an analysis of the phenotype of the genetic interaction between an ett mutation and mutations in genes involved in auxin signaling, and a similar genetic analysis in Capsella. The take-home messages are that ETT and ARF4 are functionally distinct, and that ETT operates in parallel to the canonical auxin signaling pathway in both Arabidopsis and Capsella. These conclusions are not terribly surprising, as previous biochemical and genetic studies had suggested the same thing. The transcriptomic study produced lists of genes that are specifically regulated by ARF3 and ARF4, independent of auxin. Although this information does not provide much mechanistic insight into exactly how these genes operate, it is useful for future studies. The primary weakness of this paper is that it does not address the well-known roles of ETT/ARF3 and ARF4 in leaf polarity. But this is understandable, given the research interests of the corresponding author. In short, this paper is fine. It does not represent a major advance in our understanding of the function of these genes, but it contains information that could suggest avenues for future research.

© 2025. Published by The Company of Biologists under the terms of the Creative Commons Attribution License (https://creativecommons.org/licenses/by/4.0/).
